# Unveiling Search‐Robust One‐Shot Learning Operation in FeTFT‐Based Ternary Content‐Addressable Memory

**DOI:** 10.1002/smsc.70421

**Published:** 2026-10-06

**Authors:** Hyeonjung Park, Changwoo Han, Yejoo Choi, Junseok Kim, Myeongjae Choi, Huiseong Shin, Seungjoon Jeong, Jeongheon Cho, Chaeho Kwon, Sang Min Won, Changhwan Shin

**Affiliations:** ^1^ Department of Electrical and Computer Engineering Sungkyunkwan University Suwon South Korea; ^2^ School of Electrical Engineering College of Engineering Korea University Seoul South Korea; ^3^ Semiconductor R&D Center Samsung Electronics Hwaseong South Korea; ^4^ School of Semiconductor System Engineering College of Engineering Seoul South Korea

**Keywords:** ferroelectric HZO, ferroelectric thin‐film transistors, in‐memory computing, nonvolatile memories, ternary content‐addressable memories

## Abstract

With the rapid growth of data‐centric artificial intelligence (AI), ternary content‐addressable memory (TCAM) has emerged as a promising platform for parallel in‐memory search. Here, we systematically investigate how the gate‐dielectric material and structural design of metal–ferroelectric‐metal–insulator–semiconductor (MFMIS) ferroelectric thin‐film transistors (FeTFTs) affect TCAM search operation and long‐term robustness. By comparing ZrO_2_ (11 nm), ZrO_2_ (8 nm), and HfO_2_ (8 nm) gate dielectrics, we decouple the effects of material substitution, thickness scaling, and equivalent‐oxide‐thickness (EOT)‐matched behavior. All devices show proper initial TCAM operation, with ZrO_2_ 8 nm exhibiting the highest initial sensing performance owing to its larger read current. Under repeated search cycling, however, ZrO_2_ 11 nm proves the most robust, while HfO_2_ 8 nm suffers the most severe degradation—governed by competition between electron trapping and donor‐like state creation rather than conventional ferroelectric fatigue. One‐shot classification simulations using experimentally extracted bit error rate (BER) trends confirm that device‐level degradation directly translates into accuracy loss, with ZrO_2_ 11 nm retaining the highest accuracy. Increasing the memcapacitor area ratio enlarges the memory window but degrades sensing response due to added parasitic capacitance and RC delay. These findings establish gate‐dielectric and interface design guidelines for search‐robust FeTFT‐based TCAM.

## Graphical abstract

In content‐addressable memory, stored data are searched far more often than they are written—yet search itself degrades the cell. A 2FeTFT ternary content‐addressable memory (TCAM) built on metal–ferroelectric–metal–insulator–semiconductor (MFMIS) ferroelectric thin‐film transistors reveals that gate‐dielectric choice governs how long the sensing margin survives repeated search and that this device‐level degradation directly limits one‐shot classification accuracy.

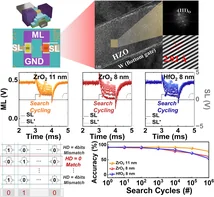

## 1 Introduction

With the rapid growth of deep learning, search operations that identify similar patterns from large‐scale datasets have become increasingly important in data‐intensive artificial intelligence (AI) workloads [1, 2, 3, 4]. In memory‐augmented neural networks and related retrieval tasks, comparing an input query with a large number of stored representations can impose substantial latency and energy overhead, thereby motivating hardware solutions that alleviate the data‐movement bottleneck of conventional von Neumann systems [1, 5, 6, 7, 8].

For this reason, ternary content‐addressable memory (TCAM) has gained significant attention as a key computing primitive for next‐generation AI hardware. TCAM performs parallel in‐memory search operations by simultaneously comparing input queries with all stored data, thereby reducing data‐movement overhead and enabling high‐throughput retrieval [5, 6, 9]. Owing to these advantages, TCAM has been widely adopted in applications requiring high‐speed parallel search, such as network routing, CPU caching, and pattern matching, and is becoming increasingly important in emerging workloads, including approximate nearest‐neighbor search and few‐shot learning [10, 11]. Among them, one‐shot learning is particularly compelling, as it enables a model to recognize and classify new concepts from only a single labeled example per class—closely mimicking the human ability to generalize from minimal experience [12, 13]. This capability is especially critical in real‐world scenarios where large annotated datasets are unavailable or costly to obtain, such as medical imaging, robotics, and personalized edge AI [14]. Because one‐shot learning relies on rapid similarity search over a stored set of class prototypes rather than iterative gradient‐based updates, it is inherently well‐suited for hardware acceleration via TCAM. The parallel associative‐search architecture of TCAM allows each incoming query to be matched against all registered class representations simultaneously and in a single inference step, enabling low‐latency, energy‐efficient classification that is difficult to achieve with conventional processor‐centric architectures. As illustrated in Figure 1A, features extracted from support‐set data by the feature extractor are first written into the TCAM array during class registration, whereas each incoming query is classified through a parallel search across the stored rows during inference. Despite these advantages, conventional complementary metal oxide semiconductor (CMOS)‐based TCAM suffers from fundamental limitations in terms of large‐scale integration and low‐power operation. The traditional static random‐acess memory (SRAM)‐based TCAM requires 16 transistors per cell, resulting in a large cell footprint as well as substantial standby and search power dissipation [15, 16]. Alternative implementations based on magnetic tunneling junctions, resistive random‐access memory, and phase‐change memory have been proposed; however, these approaches still face challenges including high write current, limited on/off current ratio, and susceptibility to device variability. These drawbacks remain critical obstacles to the realization of large‐capacity inmemory search hardware for AI applications [17].

**FIGURE 1:**
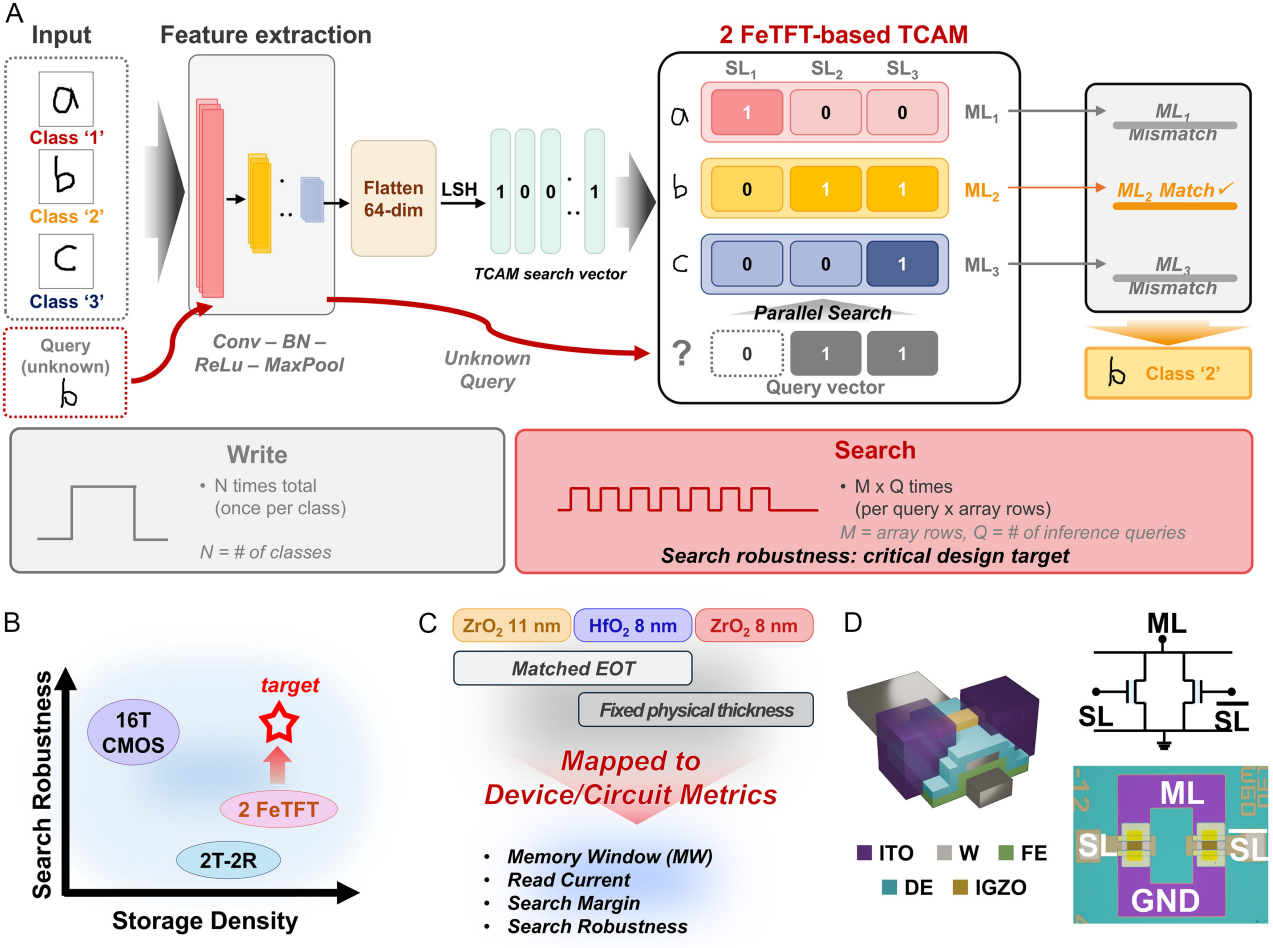
(A) Schematic illustration of FeTFT‐based TCAM for search‐intensive inference, where repeated parallel search makes search robustness a key design target. (B) Device structure, circuit schematic, and optical image of the fabricated 2T FeTFT‐based TCAM cell. (C) Comparison framework for the three dielectric conditions, ZrO_2_ 11 nm, HfO_2_ 8 nm, and ZrO_2_ 8 nm. (D) Comparison of search robustness and storage density among conventional 16T CMOS, 2T‐2R, and 2FeTFT TCAM architectures, indicating that the 2FeTFT approach offers a promising pathway toward simultaneously achieving high storage density and high search robustness.

To address these challenges, CMOS‐compatible TCAM based on HfO_2_‐derived ferroelectric materials has emerged as a promising candidate for next‐generation memory architecture (Figure 1B) [5, 6, 18, 19, 20, 21, 22, 23, 24, 25, 26]. The use of nonvolatile polarization states for information storage offers an effective route to suppressing standby power dissipation, and TCAM operation can be implemented in a two‐transistor cell configuration, which is advantageous for achieving compact and densely integrated architectures [5, 6, 18]. Among them, ferroelectric thin‐film transistors (FeTFTs) employing oxide semiconductor channels are particularly attractive, as their wide bandgap yields a low off‐state leakage current, while their amorphous nature enables good uniformity over large‐area substrates [2, 27, 28, 29, 30]. Prior work has demonstrated the feasibility of FeTFT‐based TCAMs with higher integration density and improved energy efficiency relative to conventional CMOS TCAMs, establishing FeTFTs as promising nonvolatile search devices for highly integrated memory architectures [6]. The importance of search reliability in ferroelectric‐memory‐based CAM has also been recognized in previous studies, considering that stored cells can experience repeated search operations between relatively infrequent programming events. Accordingly, prior works have investigated search endurance, search disturbance, retention, and temperature‐dependent search stability in FeFET/FeTFT‐based CAMs. However, these studies have primarily focused on demonstrating search robustness or mitigating specific reliability limitations at the device level, whereas the physical origin of progressive search‐induced degradation under practical TCAM bias conditions remains insufficiently understood [31, 32, 33].

The electrical behavior of FeTFTs is governed not only by the polarization properties of the ferroelectric layer but also by the physical and chemical properties of the gate dielectric. In particular, the gate dielectric determines the dielectric constant, interfacial charge density, subgap trap state distribution, and voltage division across the overall gate stack, thereby modulating the transfer characteristics and operational reliability of the FeTFT [34, 35, 36]. This dependence is especially pronounced in FeTFTs employing oxide semiconductor channels, where the trap state density and oxygen vacancy concentration at the gate dielectric/IGZO interface are directly linked to threshold voltage stability and device endurance. The selection of the gate dielectric therefore constitutes a critical design parameter governing the performance and reliability of metal‐ferroelectric‐metal‐insulator‐semiconductor (MFMIS) FeTFTs.

Despite this recognized importance, prior studies on FeTFT‐based TCAMs have primarily focused on device implementation and basic operational demonstrations. Systematic investigations of how dielectric‐dependent variations in FeTFT electrical characteristics translate into TCAM‐level performance metrics remain limited [5, 6, 37]. Moreover, although adjustment of the ferroelectric‐to‐dielectric area ratio in MFMIS FeTFTs is widely employed to expand the memory window, its systematic impact on circuit‐level TCAM operation remains largely unexplored. More importantly, it remains unclear how experimentally observed search degradation propagates to classification accuracy in TCAM‐based one‐shot learning [37, 38].

Accordingly, this work focuses on identifying the electrical degradation mechanisms governing cycle‐dependent search instability under TCAM‐relevant bias‐stress conditions and elucidating how the resulting device‐level electrical degradation propagates to circuit‐level sensing margin and application‐level inference performance. As shown in Figure 1C, three gate dielectric conditions, ZrO_2_ 11 nm, HfO_2_ 8 nm, and ZrO_2_ 8 nm, were selected to establish a comparison framework spanning matched‐equivalent‐oxide‐thickness (EOT) and fixed‐physical‐thickness viewpoints. Specifically, ZrO_2_ 11 nm and HfO_2_ 8 nm constitute the matched‐EOT pair, whereas HfO_2_ 8 nm and ZrO_2_ 8 nm constitute the fixed‐thickness pair. In addition, comparison between ZrO_2_ 11 nm and ZrO_2_ 8 nm enables the effect of thickness scaling within the same dielectric material system to be examined. Although wider‐bandgap dielectrics such as Al_2_O_3_ can provide a larger barrier against electron injection from IGZO, dielectric selection in an MFMIS FeTFT also requires consideration of electrostatic coupling and voltage partitioning across the gate stack. Because Al_2_O_3_ has a substantially lower dielectric constant than HfO_2_ and ZrO_2_, its incorporation would alter the insulator capacitance and voltage distribution, in addition to the band alignment. HfO_2_ and ZrO_2_ were therefore selected as single‐layer high‐k dielectrics that provide different band alignments with IGZO while maintaining strong electrostatic coupling. Each condition was evaluated in terms of memory window, read current, search margin, and search robustness, thereby establishing a hierarchical correspondence from material‐level properties through device‐level characteristics to circuit‐level TCAM behavior. Furthermore, a TCAM‐based one‐shot classification simulation incorporating experimentally extracted cycle‐dependent bit error rate (BER) trends was performed to examine how device degradation translates to classification accuracy.

In practical inference operation, write operations are performed only *N* times in total, corresponding to the number of classes registered in the TCAM array during the initial feature embedding stage. In contrast, each inference query independently triggers a complete parallel search across all *M* stored rows, and this search operation is repeated for every query presented to the system. Consequently, the cumulative number of search operations reaches *M* × *Q* over the operational lifetime of system, where *Q* denotes the total number of inference queries. Given that a single TCAM array typically stores features across a large number of classes while processing orders of magnitude more queries than registered entries, *M* × *Q* substantially exceeds *N* under any realistic operating condition. At the level of an individual cell, this asymmetry is even more pronounced: each stored cell is programmed only once during registration yet participates in a search operation for every one of the *Q* queries. Under such search‐dominant cycling, progressive degradation of the match line discharge response directly limits the sensing margin available to distinguish match from mismatch states [39]. Therefore, preserving this sensing margin during repeated search cycling is a more critical design consideration than maximizing the write‐side memory window (Figure 1A). From this perspective, our study places particular emphasis not only on the write‐side memory window characteristics at the device level but also on the degradation behavior and sensing margin retention under repeated search cycling and systematically investigates how gate dielectric material selection and structural design govern these operationally critical performance metrics.

## 2 Results and Discussion

### 2.1 Structural, Ferroelectric, and Electrical Validation of MFMIS FeTFTs

The structure of 2 FeTFT TCAM cell is shown in Figure 1D. A cross‐sectional transmission electron microscopy (TEM) image of the ZrO_2_ 11 nm device is shown in Figure 2A, where the discrete layers of the MFMIS stack—W bottom gate, hafnium zirconium oxide (HZO) ferroelectric layer, W floating metal, ZrO_2_ gate dielectric, and indium‐gallium‐zinc‐oxide (IGZO) channel—are clearly resolved with sharp interfaces. Cross‐sectional TEM images, energy‐dispersive X‐ray spectroscopy (EDS) analysis (Figure S2), and X‐ray photoelectron spectroscopy (XPS) depth profiles (Figure S3) of the ZrO_2_ 11 nm, ZrO_2_ 8 nm, and HfO_2_ 8 nm confirm that each layer was deposited with uniform and well‐defined thickness in accordance with the intended design across all three devices. To verify the ferroelectric properties of the HZO layer formed under the adopted process conditions, W/HZO/W metal‐ferroelectric‐metal (MFM) capacitors were fabricated following the same process flow as the FeTFTs. Figure 2B presents a high‐resolution cross‐sectional TEM image and the corresponding inverse fast Fourier transform (IFFT) image obtained from filtered FFT patterns of the W/HZO/W capacitor. The measured lattice spacing of 2.92 Å is consistent with the (111) reflection of the orthorhombic phase, confirming successful crystallization of HZO into the ferroelectric phase upon postmetallization annealing (PMA) [40, 41, 42]. As shown in Figure 2C, the HZO film annealed at 600 °C for 30 s exhibits a diffraction peak at 30.5°, which is associated with the orthorhombic phase, whereas the as‐deposited film shows no discernible peak, indicating the absence of crystallization prior to annealing [40]. In addition, in Figure 2D, the *P* *–* *V* and *C* *–* *V* characteristics of the W/HZO/W capacitors exhibit clear ferroelectric behavior, further confirming the robust ferroelectricity of MFM capacitors. To confirm that the EOT values of ZrO_2_ 11 nm and HfO_2_ 8 nm are matched as designed, MIM capacitors incorporating each gate dielectric were additionally fabricated and characterized; the corresponding *C*–*V* measurements are provided in Figure S4, verifying equivalent gate electrostatic conditions for these two dielectric conditions.

**FIGURE 2:**
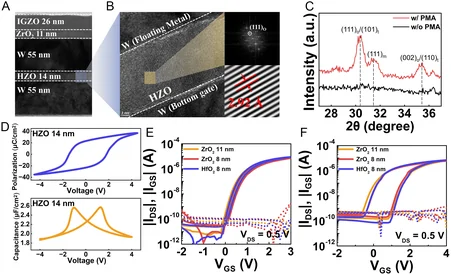
(A) Cross‐sectional TEM image of the ZrO_2_ 11 nm MFMIS FeTFT. (B) High‐resolution TEM and FFT/IFFT analysis of the W/HZO/W capacitor, confirming orthorhombic HZO. (C) XRD patterns of HZO before and after annealing. (D) *P* *–* *V* and *C* *–* *V* characteristics of the HZO MFM capacitor. (E,F) Transfer characteristics of MIS TFTs and MFMIS FeTFTs with different gate dielectrics.

Figure 2E,F show the transfer characteristics of the MIS TFTs and MFMIS FeTFTs, respectively, for all three gate dielectric conditions. All MIS TFTs exhibit clockwise hysteresis attributable to charge trapping at the dielectric/IGZO interface, whereas all MFMIS FeTFTs show stable counterclockwise hysteresis arising from ferroelectric polarization switching, confirming proper FeTFT operation across all dielectric conditions. The extracted memory window values are 1.41, 1.20, and 1.33 V for the ZrO_2_ 11 nm, ZrO_2_ 8 nm, and HfO_2_ 8 nm devices, respectively. The pulsed program/erase characteristics of the MFMIS FeTFTs obtained by applying ± 4 V, 1 ms gate pulses, are presented in Figure S5. The retention characteristics of the three MFMIS FeTFTs were additionally evaluated to verify the temporal stability of the programmed states. As shown in Figure S6, the program and erase transfer characteristics, as well as the corresponding read currents, remained nearly unchanged over the investigated retention period of up to 10^4^ s for all three gate‐dielectric conditions. These results indicate that the stored‐state separation is maintained within the measured time window and that the progressive degradation discussed below primarily reflects search‐cycling‐induced electrical instability rather than retention loss.

### 2.2 TCAM Search Operation and Search‐Cycling Degradation

The measurement configuration used to characterize TCAM cell operation is illustrated in Figure 3A; the detailed measurement procedure is provided in Figure S7. Prior to the electrical characterization of the TCAM cells and the search‐endurance measurements, all FeTFTs were subjected to 10^3^ bipolar wake‐up cycles using 4 V pulses with a pulse width of 1 ms. The wake‐up step was employed to stabilize the initial ferroelectric state and minimize the variations arising from different degrees of HZO wake‐up among three devices. The match line (ML) was first precharged to *V*_DD_ = 1 V, after which write pulses corresponding to the programmed logic states were applied to the search lines (SL and SL’) of each FeTFT, and the subsequent ML voltage transient was monitored using an oscilloscope. Distinct match and mismatch responses for each gate dielectric condition are provided in Figure S8. Figure 3B shows the evolution of mismatch‐state ML transients under repeated search cycling for ZrO_2_ 11 nm, ZrO_2_ 8 nm, and HfO_2_ 8 nm devices. The search cycling was performed for up to 10^6^ cycles among the three gate‐dielectric conditions. Within this cycling range, the three devices exhibit clearly distinguishable evolutions of Δ*V*_ML_, enabling the cell‐level sensing degradation to be quantitatively correlated with the electrical degradation of the corresponding individual FeTFTs, as discussed in the following section. Notably, an initial voltage fluctuation is observed immediately after the search–pulse transition, which is attributed to parasitic capacitive coupling associated with the measurement configuration. A detailed analysis of the origin of initial voltage fluctuation, as well as the optimization of the pulse condition and circuit configuration, is provided in Figure S9. All three devices exhibit progressive reduction in ML voltage drop with increasing cycle number, indicating gradual loss of mismatch sensing capability. The superior initial ML discharge of the ZrO_2_ 8 nm device is attributed to its higher *I*_D_ compared with the other devices as the ML discharge depth under mismatch conditions is governed by the *I*_D_ of the FeTFT in its low‐*V*_th_ state at the applied search voltage.

**FIGURE 3:**
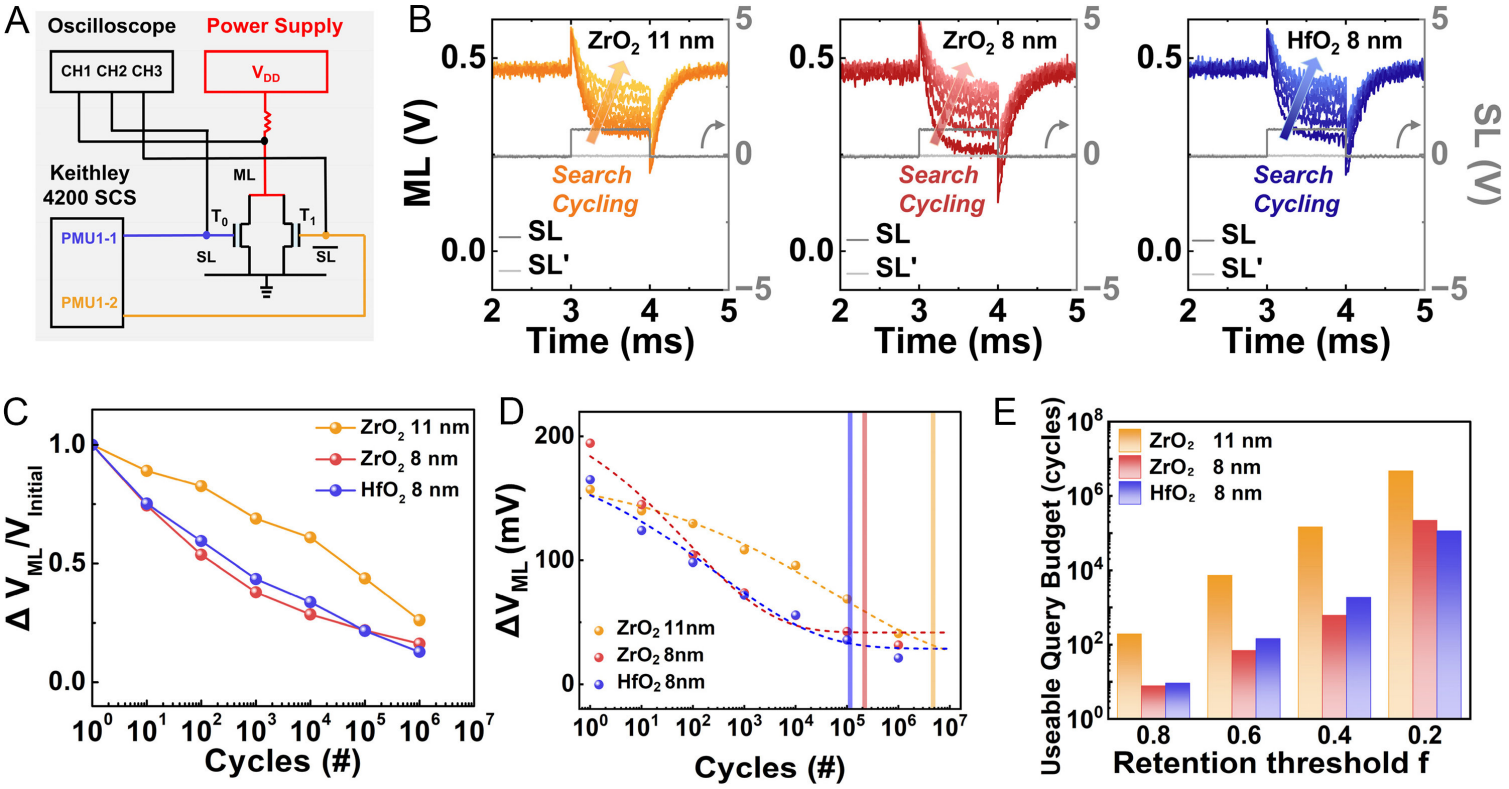
(A) Measurement setup for TCAM search characterization. (B) Transient ML responses of TCAM cells under mismatch conditions for ZrO_2_ 11 nm, ZrO_2_ 8 nm, and HfO_2_ 8 nm devices during search cycling (C) Normalized Δ*V*_ML_ as a function of search cycle number for all three devices. (D) Absolute Δ*V*_ML_ extracted at 0.5 T_read_ as a function of cycle number; vertical lines indicate the estimated operational lifetime defined by the minimum detectable search margin. (E) Useable query budget as a function of retention threshold *f* for all three devices.

Figure 3C quantitatively summarizes the normalized degradation of the ML response during search cycling in terms of Δ*V*_ML_/Δ*V*_Initial_. Δ*V*_ML_ was defined as the voltage drop from the reference level at 50% of read‐pulse duration (See Figure S10). Figure 3D plots the absolute Δ*V*_ML_ as a function of cycle number; the corresponding pulse sequence applied during the search cycling is schematically illustrated in Figure S11. This metric serves as a practical indicator of the available sensing margin under a given readout timing constraint, and the vertical lines denote the estimated operational lifetime defined by the minimum detectable search margin. Under repeated search cycling, the degradation behavior differs markedly across the three devices. The ZrO_2_ 11 nm device exhibits the most gradual reduction in Δ*V*_ML_/Δ*V*_Initial_, demonstrating the highest search robustness among the three conditions. In contrast, the ZrO_2_ 8 nm and HfO_2_ 8 nm devices show substantially faster degradation, indicating an accelerated loss of mismatch sensing capability under repeated search bias stress. Figure 3E presents the usable query budget as a function of retention threshold f, further quantifying the practical operational lifetime of each device under varying tolerance criteria.

### 2.3 Physical Origin of Search‐Induced Degradation in MFMIS FeTFTs

The degradation of TCAM operation is reflected in a reduced current difference between the match and mismatch states during the search operation, which is directly related to the *I*_D_ degradation of the FeFETs constituting the TCAM cell [39]. Therefore, to understand the origin of TCAM performance degradation, it is necessary to identify the current degradation mechanisms in MFMIS FeTFTs [43, 44]. In general, the endurance of FeTFTs is evaluated by repeatedly applying a sufficiently large gate field to induce ferroelectric polarization switching in the HZO layer, while no drain bias is applied [45, 46]. Because TCAM is inherently a search‐intensive architecture, the number of search operations experienced by a stored cell can greatly exceed the number of polarization‐switching write operations during practical operation. Accordingly, the relevant long‐term reliability concern is not limited to conventional program/erase endurance but also includes the cumulative degradation induced by repeated search bias. Specifically, the FeTFT experiences a biasing scheme in which nonswitching search pulses are repeatedly applied to the gate in the presence of a drain bias across the channel. The gate pulse employed in this work (1 V, 1 ms) is substantially lower than the coercive voltage of the 14 nm‐thick HZO layer, as determined from the *P–V* characteristics in Figure 2D. Moreover, because the externally applied gate voltage is divided between the MFM and MIS components of the MFMIS stack, only a fraction of the applied voltage is imposed across the HZO layer during actual search operation. Thus, the search pulse corresponds to a subcoercive stress condition and is not intended to induce polarization reversal. To further examine whether repeated subcoercive stressing could nevertheless induce wake‐up or fatigue of the HZO layer, we performed an additional control experiment using a discrete W/HZO (14 nm)/W capacitor fabricated with the same process that was used for the FeTFTs (Figure S12). The MFM capacitor was electrically stressed using ±1V, 1 ms bipolar pulses for up to 10^6^ cycles. As shown in Figure S12, neither the remanent polarization nor the coercive voltage exhibits a pronounced change throughout the investigated cycling range, indicating the absence of significant wake‐up or fatigue under this search‐relevant subcoercive stress condition.

Nevertheless, as shown in Figure 3C,D, search degradation is observed under practical TCAM search cycling conditions, suggesting that the degradation originates from mechanisms other than ferroelectric fatigue. The stability of the discrete MFM characteristics therefore indicates that pronounced ferroelectric wake‐up or fatigue is unlikely to be dominant origin of the observed degradation. Instead, the progressive degradation is more consistent with electrical‐stress‐induced changes in the interface between dielectric/channel, including charge trapping and defect‐state evolution. Indeed, numerous prior studies on FeTFTs with MFMIS structures have reported that device degradation is predominantly attributed to charge trapping within the gate insulator or at the gate dielectric/channel interface, rather than to changes in the polarization characteristics of the ferroelectric layer itself [43, 44, 45, 46, 47].

Given that a drain bias is inherently present during TCAM search operation, introducing a lateral electric field along the channel in addition to the vertical gate field, we designed the single‐device stress experiment to reproduce the electrical‐field condition experienced by the FeTFT during actual TCAM search operation and to separately resolve the contributions of the vertical and lateral field components to the observed degradation. Accordingly, stress cycling in this work included a drain‐biased condition (*V*_DS_ = 0.5 V) to emulate the simultaneous vertical and lateral electric fields present during practical TCAM search operation, together with a drain‐unbiased reference condition (*V*_DS_ = 0.0 V) under the same gate‐stress condition. By comparing these two cases, the degradation induced primarily by the gate‐driven vertical field could be distinguished from the additional degradation associated with the drain‐induced lateral field degradation [48]. Figure 4A,B shows the endurance characteristics of a single FeTFT under these two conditions, respectively. The *I*_D_ was measured at *V*_GS_ = 0.5 V throughout cycling. Note that this comparison also allows ferroelectric wake‐up to be excluded as the origin of the cycling‐induced changes discussed below. Wake‐up of the HZO layer is driven solely by the vertical field established across the ferroelectric by the gate bias and is therefore independent of the drain bias. If wake‐up were responsible, an identical early‐stage current increase would be expected at *V*_DS_ = 0 V and *V*_DS_ = 0.5 V. In practice, however, the early current increase is observed exclusively at *V*_DS_ = 0.5 V and only for the ZrO_2_ 11 nm device (Figure 4A,B). This *V*_DS_‐dependent behavior cannot be reconciled with HZO wake‐up and instead indicates that the current increase is governed by the lateral electric field.

**FIGURE 4:**
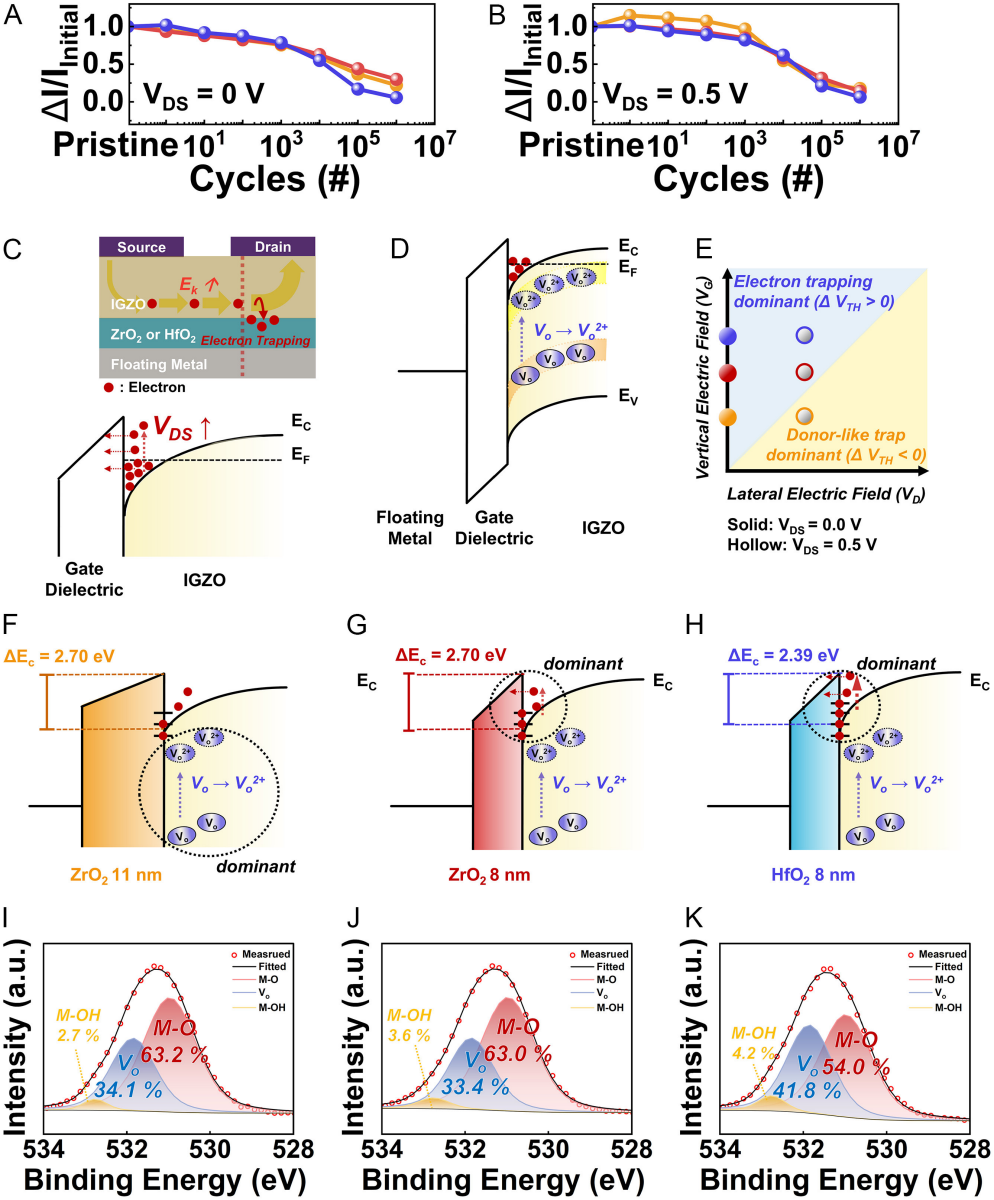
(A,B) Cycling‐induced change in normalized read current at *V*_DS_ = 0 and 0.5 V. (C,D) Schematics of electron trapping and donor‐like state creation. (E) Competition between the two mechanisms as a function of vertical and lateral electric fields. (F–H) Device‐specific band‐diagram interpretation. (I–K) Deconvoluted O 1s XPS spectra of IGZO on the three dielectrics.

When cycling was performed without drain bias, all three devices exhibited current degradation from the early stage. Among them, the HfO_2_ 8 nm device showed the most pronounced reduction in read current, indicating relatively poor degradation tolerance. Under a drain bias of 0.5 V, a distinctly different behavior emerged. While the ZrO_2_ 8 nm and HfO_2_ 8 nm devices continued to exhibit current degradation, the ZrO_2_ 11 nm device showed a slight current increase during the early stage of cycling, demonstrating comparatively robust behavior.

To understand these distinct behaviors, it is necessary to consider the physical origins of current degradation in the IGZO channel. Previous studies have established that drain current (*I*_D_) degradation in IGZO‐channel TFTs can be attributed to two competing mechanisms: electron trapping in the gate dielectric or at the dielectric/channel interface and donor‐like state creation within the channel [49, 50]. Because these two mechanisms induce *V*_th_ shifts in opposite directions—positive and negative, respectively—the observed degradation behavior reflects their relative dominance under a given bias condition.

The first mechanism is electron trapping, which occurs when electrons are captured by trap states in the gate dielectric or at the dielectric/IGZO interface under a gate‐induced vertical electric field. The trapped charges partially screen the gate field, resulting in a positive shift in *V* ^th^ and, consequently, a reduction in the *I*_D_ [49]. When a drain bias is additionally applied, the lateral electric field further promotes electron trapping, as electrons traversing the channel acquire additional kinetic energy and are more readily injected into trap states (Figure 4C) [51].

The second mechanism is donor‐like state creation induced by the lateral local electric field generated by the drain bias (Figure 4D). Under a sufficiently high local electric field, electrons accelerated in the lateral direction can break weak oxygen bonds, such that neutral oxygen vacancies (*V* *_O_*) may undergo impact ionization to form additional ${V_{O}}^{2\text{+}}$ states [50]. The generation of ${V_{O}}^{2\text{+}}$ can be expressed as:

(1) $$V_{O}+e^{-}\to {V_{O}}^{2\text{+}}+3e^{-}$$

This process increases the density of shallow donor‐like subgap states near the conduction band edge, thereby raising the free‐electron concentration and causing an increase in *I*_D_. As cycling continues, electron trapping progressively becomes dominant, leading to a transition from an initial negative *V*_th_ shift to a positive one. Figure 4E schematically summarizes the competition between these two mechanisms as a function of the relative strengths of the vertical and lateral electric fields.

The degradation behaviors observed in FeTFTs can thus be interpreted in terms of the competing mechanisms described above, as schematically illustrated in Figure 4F–H. When cycling was performed at *V*_DS_ = 0 V, all three devices were governed by electron trapping, showing a positive *V*_th_ shift and a monotonic decrease in *I*_D_. At *V*_DS_ = 0.5 V, only the ZrO_2_ 11 nm device exhibited a pronounced donor‐like‐state‐related current increase in the early stage of cycling, after which electron trapping became dominant as cycling proceeded. The ZrO_2_ 8 nm and HfO_2_ 8 nm devices remained electron‐trapping‐dominated throughout.

Comparing the EOT‐matched ZrO_2_ 11 nm and HfO_2_ 8 nm devices reveals that similar gate controllability does not translate to similar degradation behavior. The HfO_2_ 8 nm device exhibits more severe *I*_D_ degradation, which is attributed to material‐dependent properties rather than field strength alone. Specifically, HfO_2_ has a smaller conduction band offset with IGZO than ZrO_2_, facilitating electron injection into the dielectric or interfacial trap states. Furthermore, XPS O 1s peak analysis confirms that the HfO_2_‐based device possesses a higher density of oxygen‐vacancy‐related trap states, indicating a larger trap reservoir and consequently greater susceptibility to electron trapping (Figure 4I–K) [52, 53]. Complementary In 3d XPS analysis was conducted to independently assess the oxygen‐deficiency‐related chemical states in IGZO channel (Figure S13). The HfO_2_‐based condition exhibited a higher fraction of the oxygen‐vacancy‐associated In component than the two ZrO_2_‐based conditions, consistent with the O 1s analysis. The agreement between the O 1s and In 3d analyses supports a more pronounced oxygen‐deficiency‐related defect environment in IGZO formed on HfO_2_, consistent with its greater susceptibility to electron trapping.

Comparing the physically thickness‐matched ZrO_2_ 8 nm and HfO_2_ 8 nm devices leads to the same conclusion: HfO_2_ has a smaller dielectric constant than ZrO_2_, and its conduction‐band offset with IGZO is smaller, allowing electrons to be injected more readily into the dielectric and interfacial trap states. Electron trapping is therefore more pronounced in the HfO_2_ device. Finally, comparing the ZrO_2_ 11 nm and ZrO_2_ 8 nm devices isolates the effect of dielectric thickness. The thinner ZrO_2_ 8 nm layer produces a stronger vertical electric field under the same gate bias, promoting electron injection from an early stage of cycling and making electron trapping dominant throughout. The thicker ZrO_2_11 nm device, by contrast, experiences a weaker vertical field, suppressing electron trapping and allowing donor‐like state creation to manifest in the early cycling stage.

In summary, the HfO_2_ 8 nm device exhibits the most electron‐trapping‐dominant behavior due to its smaller conduction band offset and higher trap density, resulting in the largest *I*_D_ degradation among the three devices. These results demonstrate that TCAM search‐induced degradation in FeTFTs is fundamentally governed by the interplay between dielectric material properties, thickness‐dependent field distribution, and the resulting competition between electron trapping and donor‐like state creation, rather than being determined solely by the magnitude of the applied read bias. To further examine the temperature dependance of the proposed degradation mechanism, search‐equivalent single‐FeTFT cycling measurements were additionally performed at 85 °C under the same drain‐biased stress condition (*V*_DS_ = 0.5 V), as shown in Figure S14. At 25 °C, an initial increase in the normalized read current was primarily observed for the ZrO_2_ 11 nm device, whereas the ZrO_2_ 8 nm and HfO_2_ 8 nm devices exhibited trapping‐dominant current degradation from the early stage. In contrast, at 85 °C, all three devices exhibited a slight initial current increase before the subsequent current degradation. This temperature‐dependent transition is consistent with the enhanced contribution of oxygen‐vacancy‐related donor‐like states at elevated temperature, as reported for a‐IGZO TFTs under bias‐temperature stress, which shifts the early‐stage competition toward donor‐like state creation. With continued cycling, however, accumulated electron trapping progressively becomes dominant, leading to a subsequent decrease in read current [48, 51]. These results further support that the search‐induced degradation is governed by the competition between donor‐like state creation and electron trapping, whose relative contributions vary with temperature.

In addition to these material‐ and thickness‐dependent effects, it is worth noting that structural design strategies commonly adopted in MFMIS FeTFTs, such as tuning the MFM‐to‐MIS area ratio to enlarge the memory window through more favorable voltage division, are not necessarily beneficial for TCAM operation. Although such area‐ratio engineering can improve ferroelectric switching characteristics at the individual‐device level, it may simultaneously increase parasitic capacitance and delay the dynamic match‐line response, thereby making it less suitable for search‐oriented TCAM operation. This structural trade‐off is briefly discussed in Supporting Information (Figure S15). Taken together, these results establish three electrical design considerations for improving the search robustness of oxide‐channel MFMIS FeTFT‐based TCAMs. First, the gate dielectric should provide a sufficiently large conduction‐band offset with the semiconductor channel to suppress carrier injection into dielectric and interfacial trap states. Second, the dielectric/channel interface should be engineered to minimize electrically active trap states and oxygen‐deficiency‐related defects that promote charge trapping during repeated search bias. Third, the dielectric thickness and capacitance should be co‐optimized to limit the vertical electric field across the MIS stack while maintaining sufficient gate control. These results indicate that search robustness cannot be optimized solely through the initial memory window or gate capacitance but requires simultaneous consideration of band alignment, defect characteristics, and search‐bias‐induced electric‐field distribution.

### 2.4 Impact of Device Degradation on One‐Shot Classification Accuracy

To examine how the experimentally observed search characteristics propagate to inference‐level performance, a TCAM‐based one‐shot classification simulation was performed using a locality sensitive hashing (LSH)‐assisted associative‐search framework. TCAM‐based parallel search has been established as an effective hardware primitive for one‐shot learning, and hardware memory‐augmented learning with hashing and similarity search has also been experimentally demonstrated in prior works [1, 5, 54]. In this simulation, support and query images from the Omniglot evaluation dataset were first embedded into 64‐dimensional feature vectors using a four‐layer convolutional neural network, and the extracted features were then converted into binary hash codes through random‐projection‐based locality‐sensitive hashing [12, 55]. The binary support codes were stored in the TCAM array, and an unknown query was classified by parallel comparison with all stored rows, as illustrated in Figure 5A. For each stored row, the number of sensed mismatches serves as a measure of Hamming distance (HD) between the query and the stored code, and the class corresponding to the row with the minimum HD—indicating the closest match—is selected as the predicted class. For each bit, the match or mismatch sensing result was determined by Monte Carlo sampling using the experimentally extracted match and mismatch Δ*V*_ML_ values together with a Gaussian variation term, followed by application of a sensing threshold *θ*. The class corresponding to the row with the minimum number of sensed mismatches was then selected as the prediction.

**FIGURE 5:**
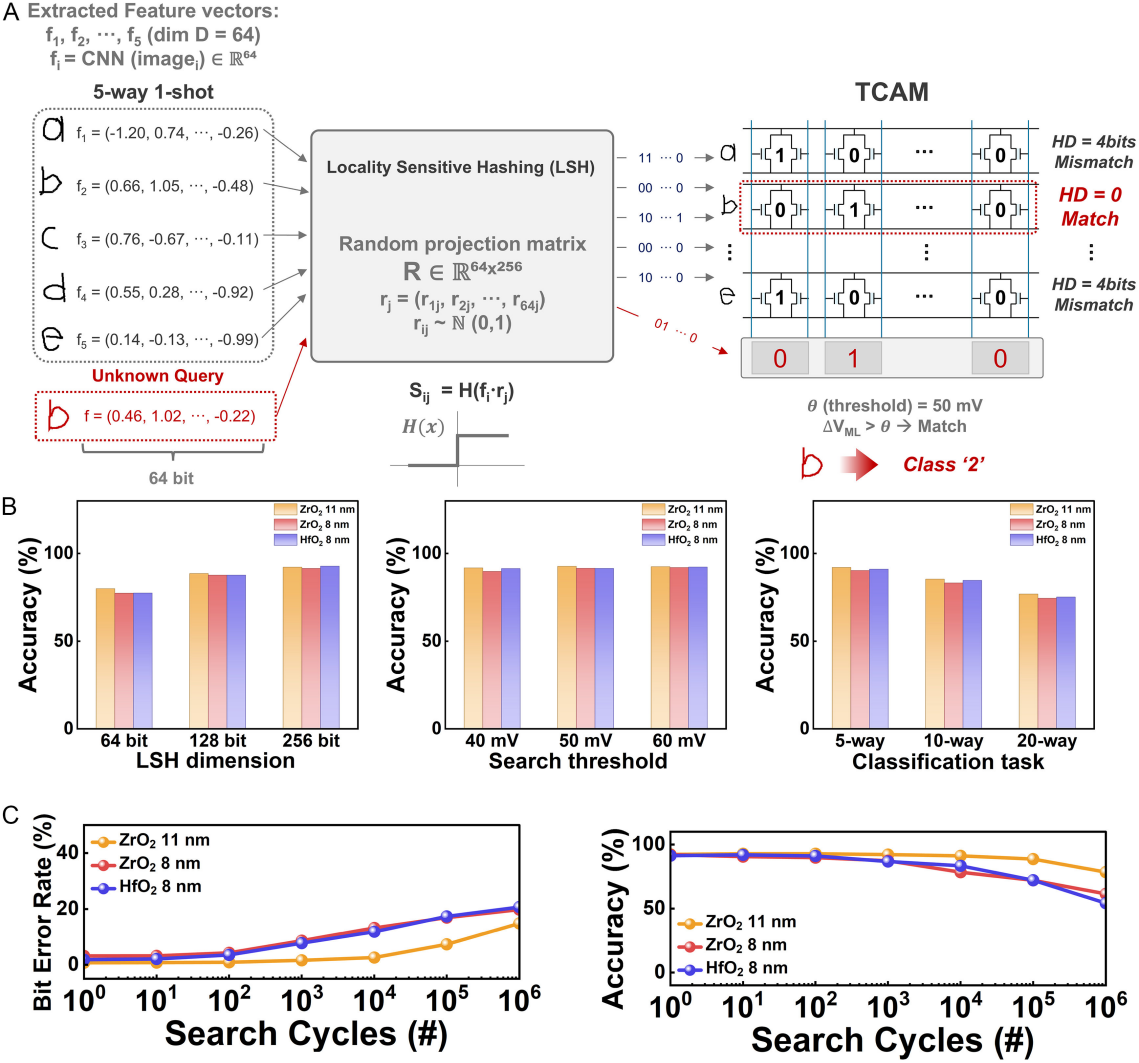
(A) Schematic of the LSH‐CAM‐based one‐shot classification pipeline. Feature vectors extracted by CNN are projected into binary hash codes via locality sensitive hashing (LSH) and stored in the TCAM array; an unknown query is classified by identifying the best‐matching stored entry based on the ML discharge response. (B) Classification accuracy in the pristine state as a function of LSH dimension, search threshold, and classification task difficulty for ZrO_2_ 11 nm, ZrO_2_ 8 nm, and HfO_2_ 8 nm devices. (C) Evolution of bit error rate (left) and one‐shot classification accuracy (right) as a function of search cycle number for all three devices.

Figure 5B shows the one‐shot classification accuracy in the pristine state as a function of LSH dimension, search threshold, and classification task difficulty. All three dielectric conditions exhibit comparable accuracy over the explored conditions, indicating that the initial device‐level differences do not strongly perturb inference performance before cycling. To further evaluate the effect of search degradation, the experimentally extracted cycle‐dependent search characteristics of each device were incorporated into the Monte Carlo sensing model, from which the bit‐error rate and the resulting one‐shot classification accuracy were calculated at each search cycle. As shown in Figure 5C, the bit‐error rate increases with search cycle number for all three devices, and the classification accuracy correspondingly decreases under a five‐way one‐shot with a 256‐bit LSH code and a sensing threshold of 50 mV. Among them, the ZrO_2_ 11 nm device maintains the lowest bit‐error rate and the highest classification accuracy throughout cycling, whereas the ZrO_2_ 8 nm and HfO_2_ 8 nm devices exhibit faster error growth and a steeper accuracy drop. These results demonstrate that the experimentally identified device‐level search robustness directly determines inference reliability in TCAM‐based one‐shot learning. The key device and TCAM performance metrics for the three gate‐dielectric conditions, including the memory window, Δ*V*_ML_, and one‐shot classification accuracy before and after search cycling, are summarized in Table 1.

**TABLE 1:** Summary of the initial device characteristics and TCAM search performance for the three gate‐dielectric conditions. The memory window (MW), initial match‐line voltage difference (Δ*V*_ML_), and one‐shot classification accuracy are compared with the corresponding Δ*V*_ML_ and classification accuracy after search cycles.

Gate dielectric	MW, V	**Δ*V***_**ML**_ **Δ*V***_**ML,Initial**_ **at 10** ^**6**^ **cycles, %**	**Initial** **accuracy,** **%**	**Accuracy** **at 10** ^**6**^ **cycles, %**
ZrO_2_ 11 nm	1.41	26.12	92.3	78.5
ZrO_2_ 8 nm	1.20	16.33	92.1	61.5
HfO_2_ 8 nm	1.33	12.79	91.3	54.3

## 3 Conclusion

In this study, we systematically investigated how the material and thickness of the gate dielectric affect the search operation and degradation behavior of 2FeTFT TCAMs based on HZO‐based MFMIS FeTFTs. Three gate‐dielectric conditions, ZrO_2_ 11 nm, ZrO_2_ 8 nm, and HfO_2_ 8 nm, were compared to separately identify the thickness‐scaling effect within the same material, the material‐substitution effect at the same physical thickness, and the intrinsic material‐dependent effect under EOT‐matched conditions. Although all three devices exhibited proper initial 2FeTFT TCAM operation, their initial search performance and stability under repeated search cycling showed distinct trends. The ZrO_2_ 8 nm device exhibited the best initial sensing performance because of its higher read current in the low‐*V*_th_ state. In contrast, under repeated search cycling, the ZrO_2_ 11 nm device showed the smallest reduction in Δ*V*_ML_ and thus the highest search robustness, whereas the HfO_2_ 8 nm device exhibited the fastest and most severe degradation. Current‐degradation analysis at the single‐FeTFT level revealed that degradation under practical TCAM search conditions is determined not simply by polarization‐switching endurance but by the simultaneous application of gate read pulses and drain bias. Without drain bias, all three devices showed positive Vth shifts and reduced *I*_D_, indicating electron‐trapping‐dominant degradation. Under *V*_DS_ = 0.5 V, corresponding to the actual search condition, electron trapping induced by the vertical field competed with donor‐like state creation promoted by the lateral field, and this competition governed the degradation behavior of each device. The HfO_2_ 8 nm device showed the most severe current degradation and sensing loss, which is attributed to its smaller conduction‐band offset and higher trap density. One‐shot classification simulation further confirmed that these device‐level degradation trends directly translate into classification accuracy loss. In the pristine state, all three devices achieved comparable accuracy across varying LSH dimensions, search thresholds, and task difficulties, whereas under repeated search cycling, the ZrO_2_ 11 nm device retained the highest accuracy, exceeding ~80% even at 10^6^ cycles, while the HfO_2_ 8 nm device showed the steepest accuracy drop. Area‐ratio‐dependent structural characteristics are provided in Supporting Information. These results indicate that FeTFT‐based TCAMs should be optimized from the perspective of actual search operation and search‐induced degradation rather than solely by static memory window or device‐level figures of merit.

## 4 Experimental Section

All devices were fabricated on p‐Si substrates. To electrically isolate individual devices, a 300 nm thick SiO_2_ layer was formed by wet oxidation. A 55 nm thick W bottom gate was deposited by reactive DC sputtering at 100 W. The ferroelectric HZO layer was subsequently deposited at 280 °C by plasma‐enhanced atomic layer deposition (PEALD) using tetrakis (ethylmethylamino) hafnium (TEMAH) and tetrakis (ethylmethylamino) zirconium (TEMAZ) as precursors and H_2_O as the reactant. After HZO deposition, a 55 nm‐thick W floating metal layer was deposited using reactive DC sputtering at 100 W. The devices were subjected to PMA at 600 °C for 30 s under N_2_ ambient to induce crystallization into the ferroelectric phase. Subsequently, ZrO_2_ (11 nm), ZrO_2_ (8 nm), and HfO_2_ (8 nm) were deposited as gate dielectric layers. Note that ZrO_2_ (11 nm) and HfO_2_ (8 nm) have same equivalent oxide thickness (EOT) values. All dielectric layers were grown at 280 °C by PEALD using TEMAZ for ZrO_2_ and TEMAH for HfO_2_, with H_2_O as the reactant in both cases. A 26 nm thick IGZO active layer was then deposited at room temperature by RF sputtering at 75 W using an alloy target with an In_2_O_3_:Ga_2_O_3_:ZnO molar ratio of 1:1:1, followed by postdeposition annealing (PDA) at 300 °C for 30 min in air to activate the channel. Finally, ITO source/drain electrodes were deposited at room temperature by RF Sputtering at 100 W using an alloy target composed of 90 wt% In_2_O_3_ and 10 wt% SnO_2_, defining a channel length (L) and width (W) of 30 and 60 μm, respectively. In TCAM cell, the ITO source/drain electrodes of each FeTFT were commonly shared, such that the two drain electrodes form the ML and the two source electrodes form the ground line.

## Funding

This study was supported by the Ministry of Trade, Industry, and Energy (2410012306, 02305570, and 25069‐15FC) and the National Research Foundation of Korea (RS‐2023‐00260527).

## Conflicts of Interest

The authors declare no conflicts of interest.

## Supporting information

Supplementary Material

## Data Availability

The data that supports the findings of this study are available in the Supporting Information of this article.
