# Supplementary material for: Unveiling Search‐Robust One‐Shot Learning Operation in FeTFT‐Based Ternary Content‐Addressable Memory
Source: Small Sci. 2026 Oct 6;6(10):e70421. doi: 10.1002/smsc.70421 (PMC13643206; doi:10.1002/smsc.70421)
Supplement: Supplementary file 1 [file SMSC-6-e70421-s001.pdf]

Supporting Information

Unveiling Search-Robust One-shot Learning Operation in FeTFT-Based Ternary Content-Addressable Memory

Hyeonjung Park [1‡], Changwoo Han [2‡], Yejoo Choi [3‡], Junseok Kim [2‡], Myeongjae Choi [4], Huiseong Shin[2], Seungjoon Jeong[2], Jeongheon Cho[2], Chaeho Kwon[2], Sang Min Won[1], and Changhwan Shin[2]*

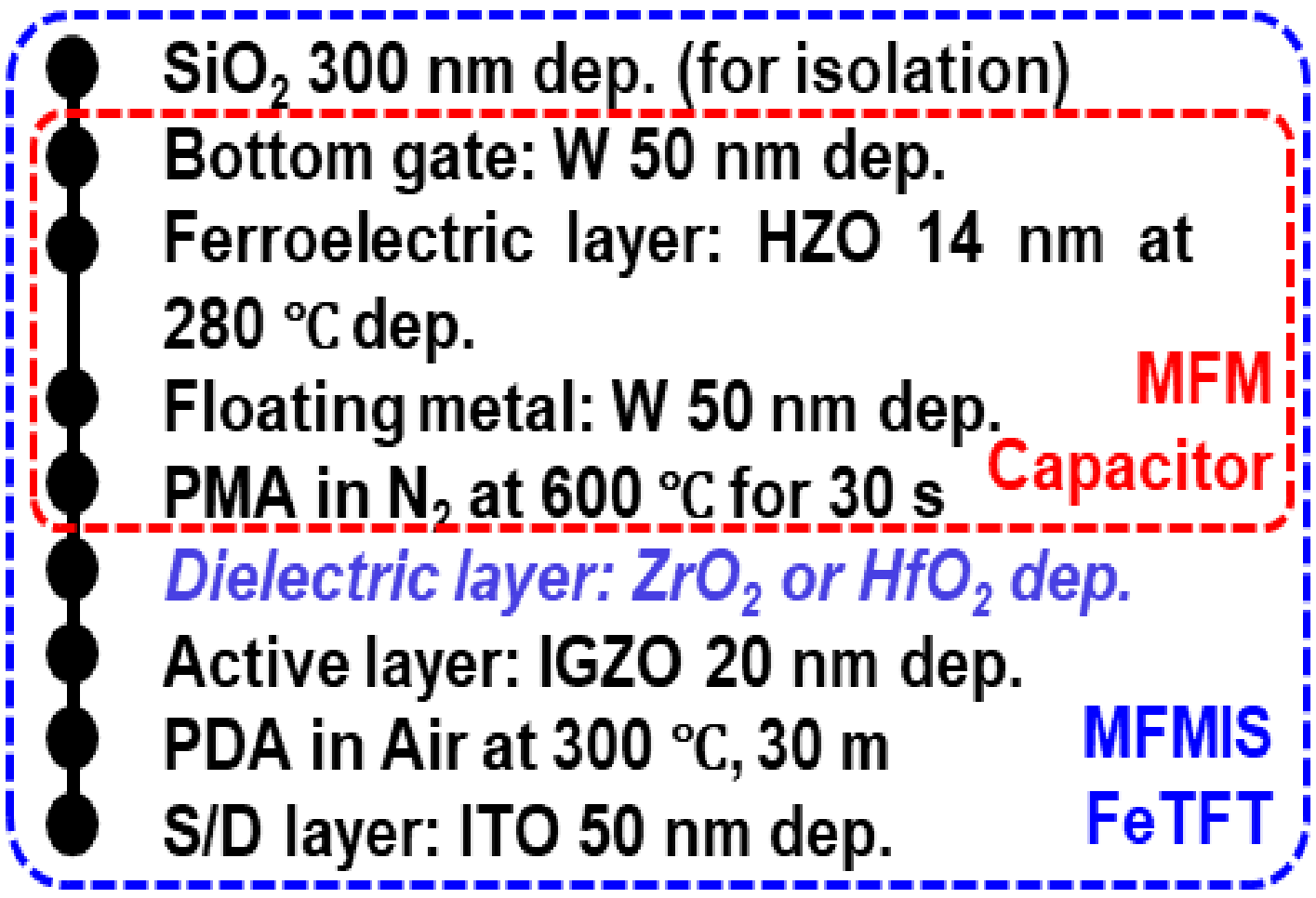

Figure S1. Process flow of fabricated MFMIS FeTFTs and MFM capacitors in this work

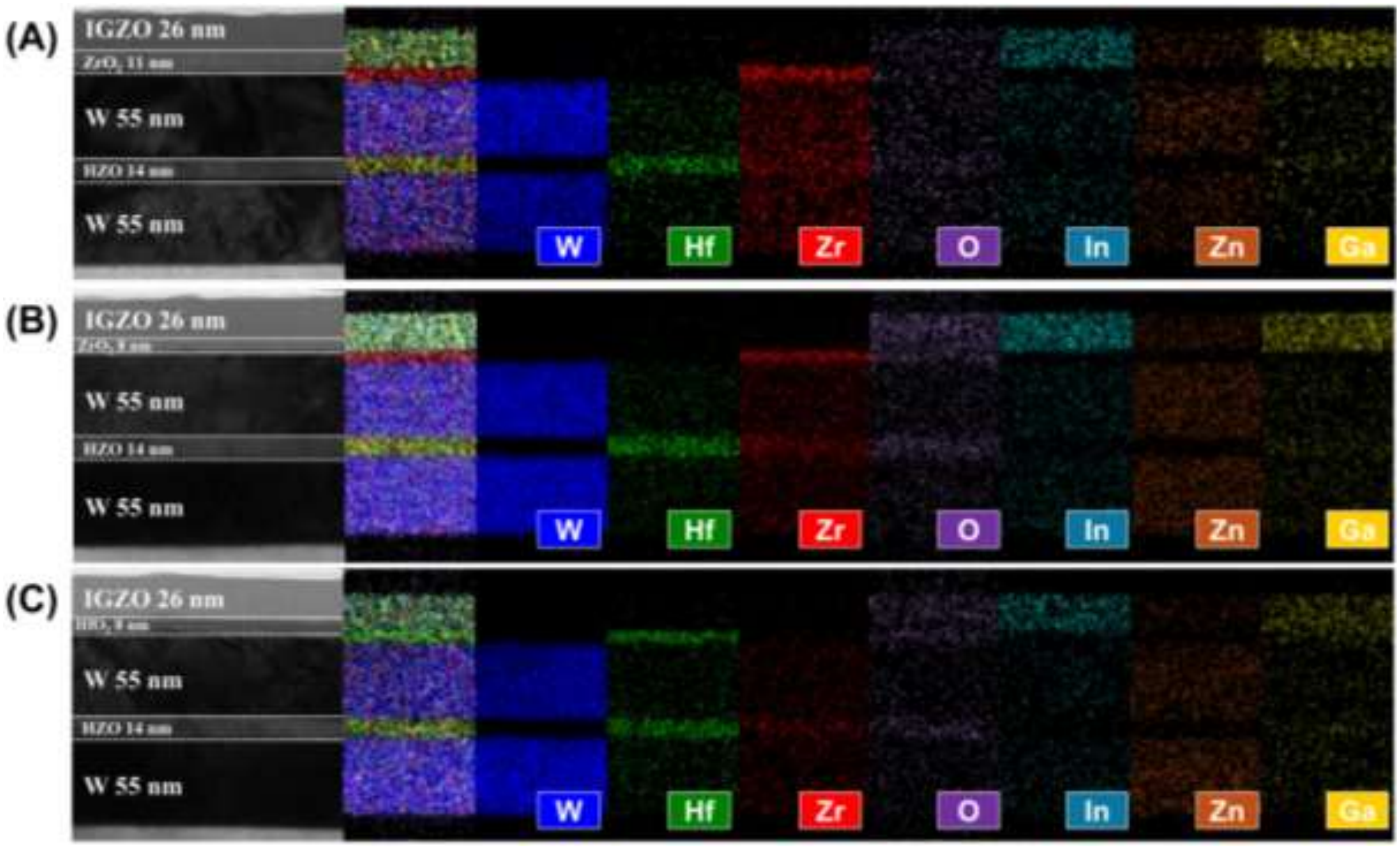

Figure S2. Cross-sectional TEM images and corresponding EDS elemental maps (W, Hf, Zr, O, In, Zn, Ga) for the three gate-dielectric: **(A)** ZrO_2 11 nm, **(B)** ZrO_2 8 nm, and **(C)** HfO_2 8 nm devices. All devices share the common stack structure of IGZO (26 nm) / gate dielectric / W floating metal (55 nm) / HZO (14 nm) / W bottom gate (55 nm). The elemental maps confirm the well-defined spatial distribution of each constituent layer, with no evidence of inter-layer intermixing, verifying that all intended layers were deposited as designed.

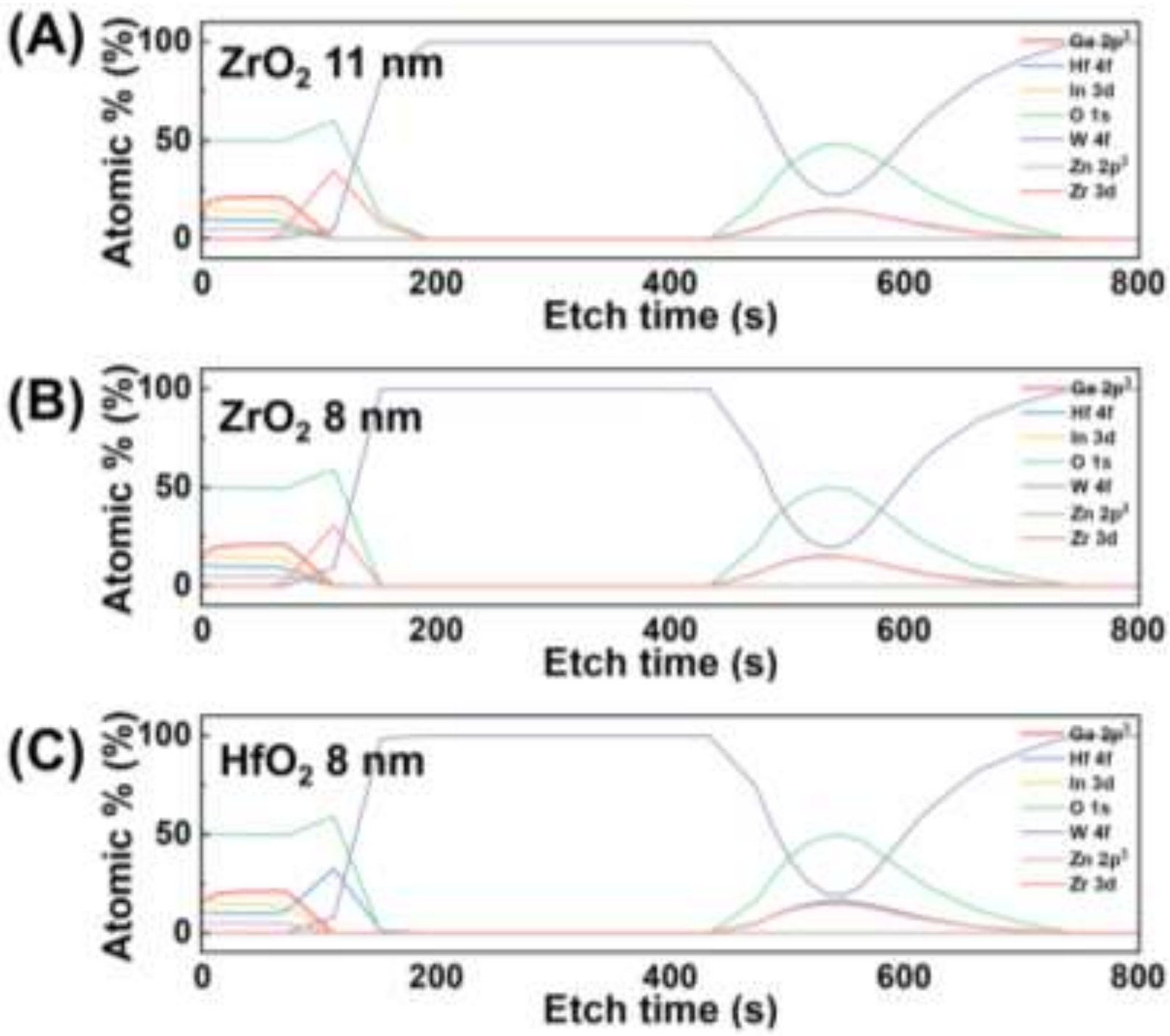

Figure S3. Elemental atomic concentration profiles as a function of ion etch time for **(A)** ZrO_2 11 nm, **(B)** ZrO_2 8 nm, and **(C)** HfO_2 8 nm devices. The sequential and well-separated emergence of characteristic elemental confirms that each constituent layer is spatially well-defined with no evidence of inter-layer intermixing, verifying the successful deposition of the intended device stack.

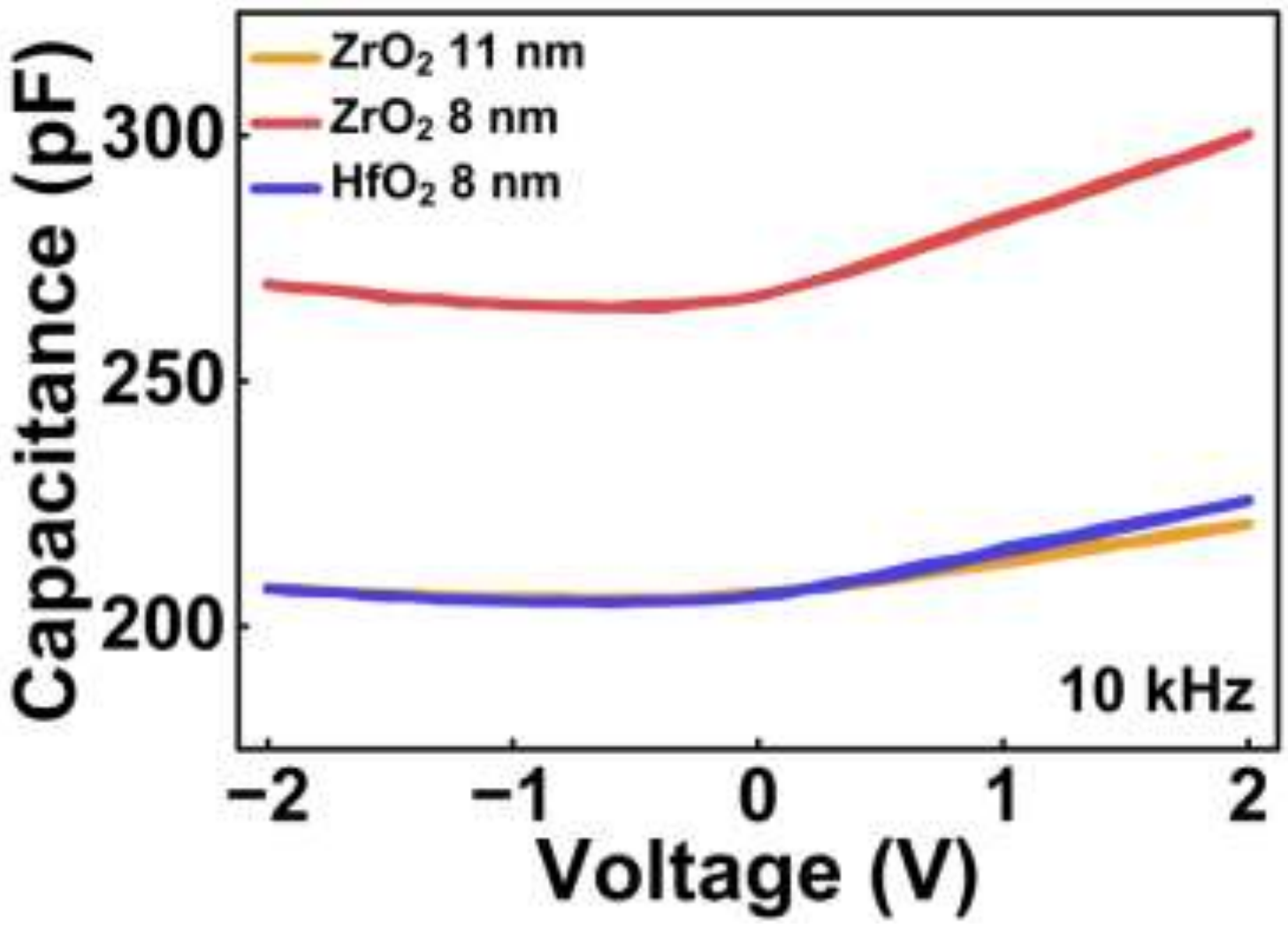

Figure S4. Capacitance–voltage (C–V) characteristics of the three gate-dielectric variants measured on the MIM capacitor structures. The ZrO_2 11 nm and HfO_2 8 nm devices exhibit nearly identical capacitance values across the full voltage sweep, confirming that the two devices are EOT-matched despite differing in dielectric material and physical thickness. The lower capacitance of the ZrO_2 8 nm device reflects its reduced physical thickness relative to ZrO_2 11 nm within the same material.

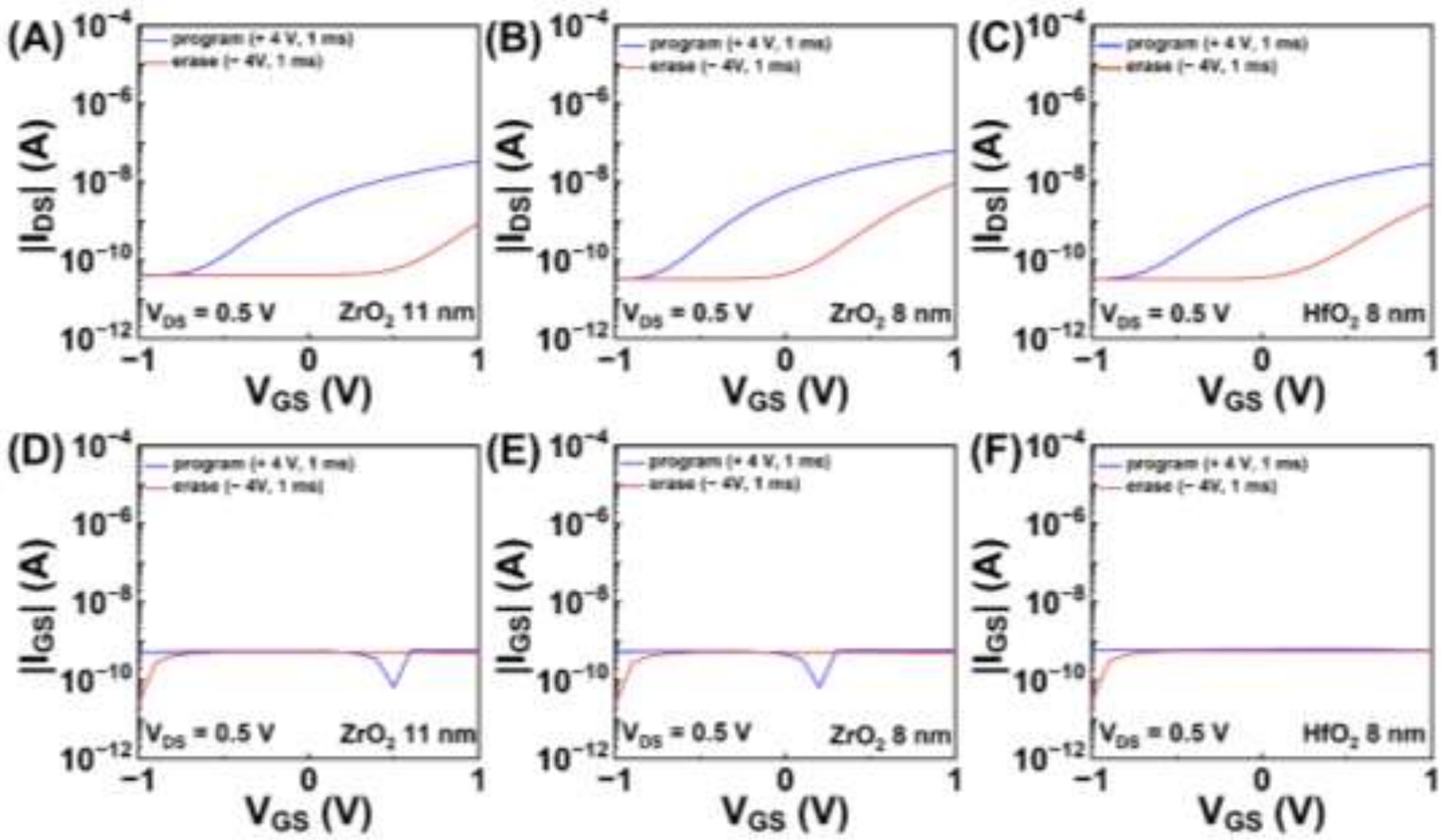

Figure S5. Memory characteristics of the MFMIS FeTFTs measured by program/erase pulses. **(A–C)** Transfer curves measured after program (+ 4 V, 1 ms) and erase (− 4 V, 1 ms) pulses, with the read sweep limited to −1 to 1 V (V_{DS} = 0.5 V) to avoid disturbing the stored polarization. **(D–F)** Corresponding gate leakage currents.

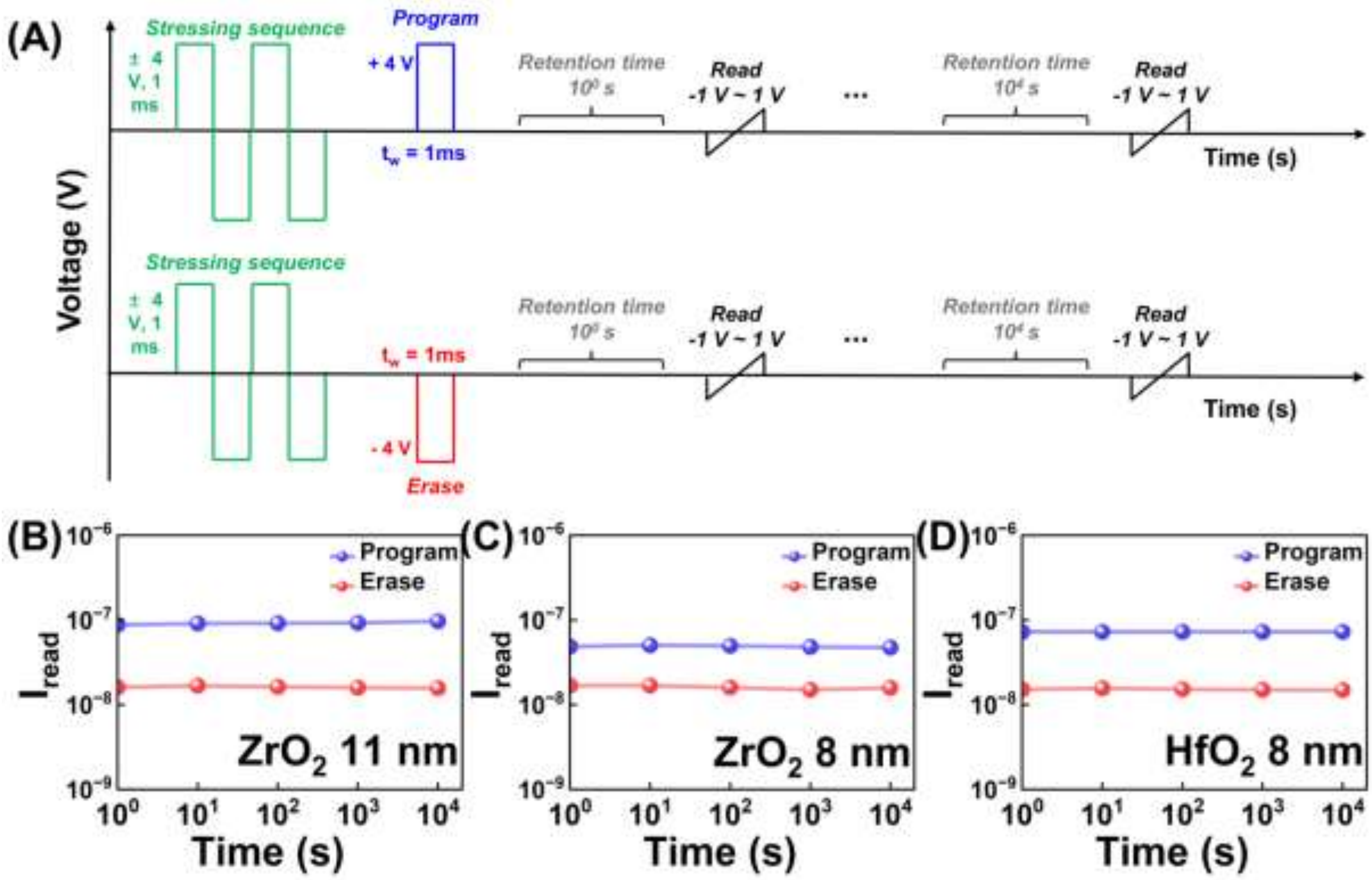

Figure S6. Retention characteristics of the MFMIS FeTFTs with different gate dielectrics. (A) Schematic of the retention-measurement sequence. Prior to retention characterization, the FeTFTs were preconditioned using 10^3 bipolar ±4 V pulses with a pulse width of 1 ms. The program and erase states were subsequently set using +4 V and −4 V, 1 ms gate pulses, respectively. After state programming, the devices were maintained without additional gate stress for selected retention times from 10^0 to 10^4 s, followed by transfer-characteristic measurements over V_{GS} = -1 V to 1 V at V_{DS} = 0.5 V. (B–D) Read currents extracted at V_{GS} = 0.5 V and V_{DS} = 0.5 V as a function of retention time for the (B) ZrO_2 11 nm, (C) ZrO_2 8 nm, and (D) HfO_2 8 nm devices, respectively. The program and erase read currents remain nearly unchanged over the investigated retention period, maintaining a clear separation between the two memory states.

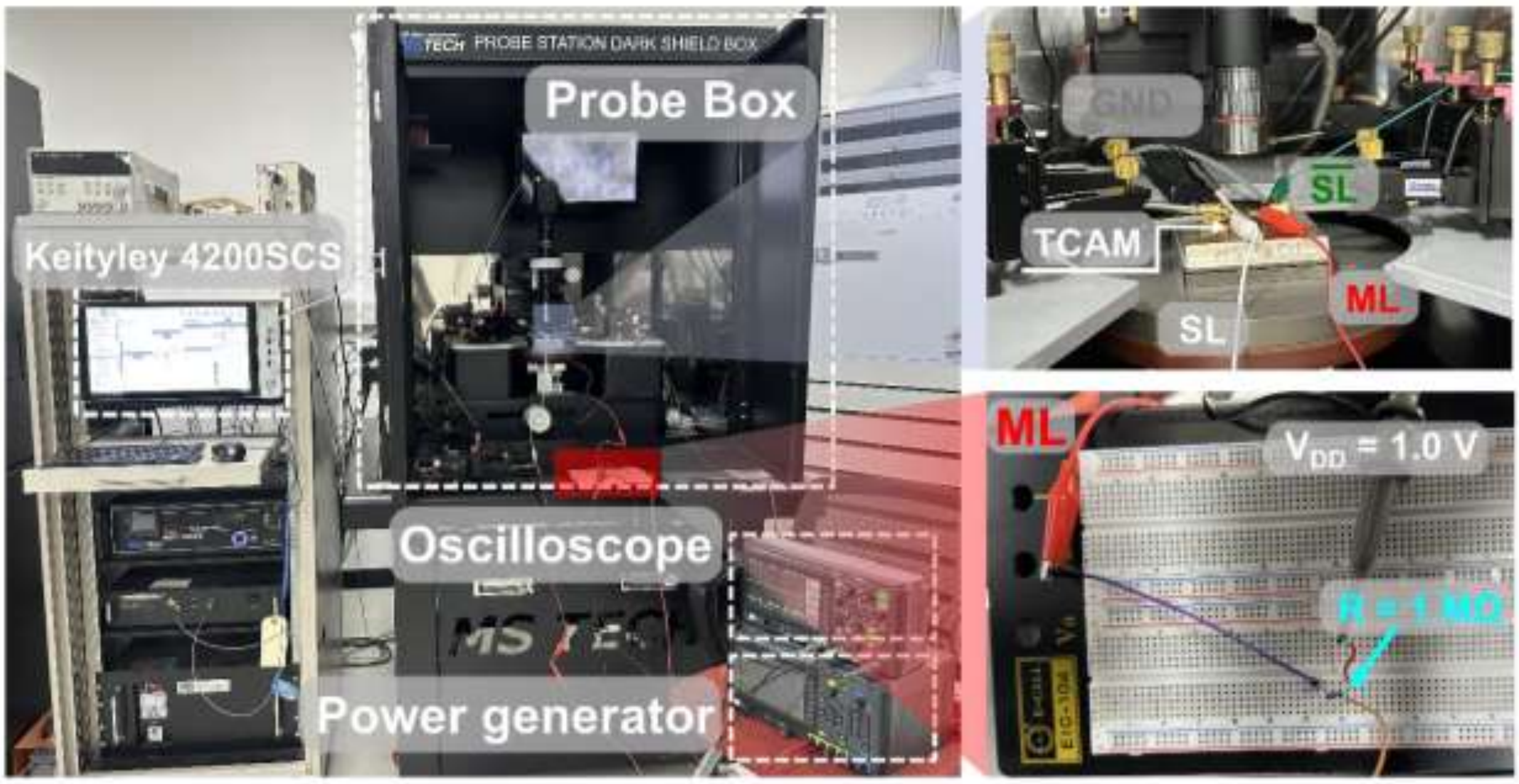

Figure S7. Experimental setup for TCAM cell characterization, including the probe station, Keithley 4200-SCS parameter analyzer, oscilloscope, and external power generator. During the measurement, the ML was biased through an external 1 MΩ resistor with V_{DD} = 1.0 V, while write and search pulses were applied to the search lines (SL and SL′) via PMU. The transient ML response was monitored by the oscilloscope, establishing an effective drain bias of approximately $V_{DS} \approx 0.5$ V during the search operation.

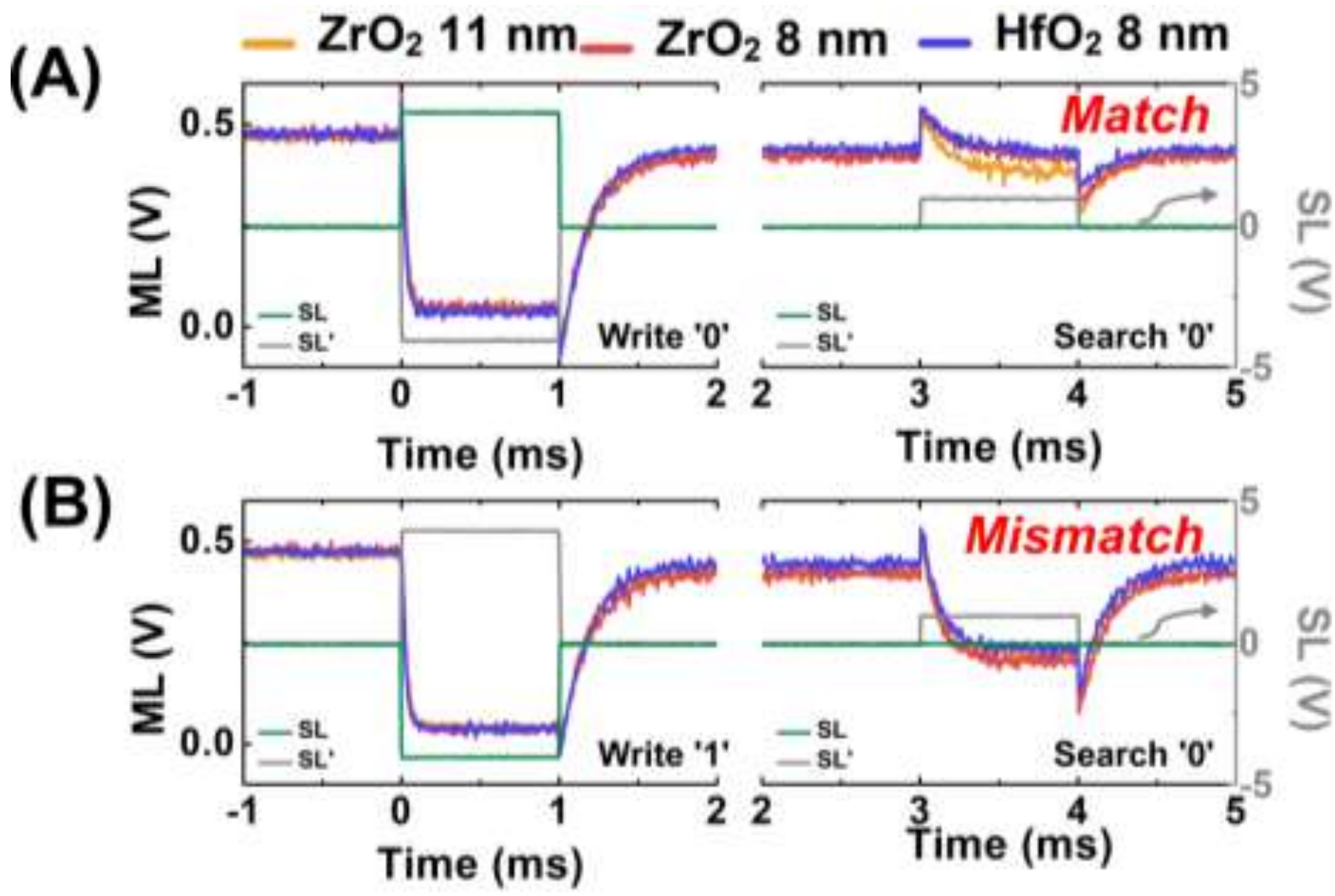

Figure S8. Time-domain matchline (ML) voltage responses during write and search operations for ZrO_2 11 nm, ZrO_2 8 nm, and HfO_2 8 nm based TCAM cells. **(A)** Write '0' operation: ML discharges upon application of a write pulse via search lines (SL, SL'), then recovers to the precharged level. **(B)** Write '1' operation: ML exhibits analogous discharge behavior. In both cases, the subsequent Search '0' operation yields clearly distinguishable ML voltage responses between match and mismatch states, validating the ternary content-addressable memory functionality of the fabricated devices.

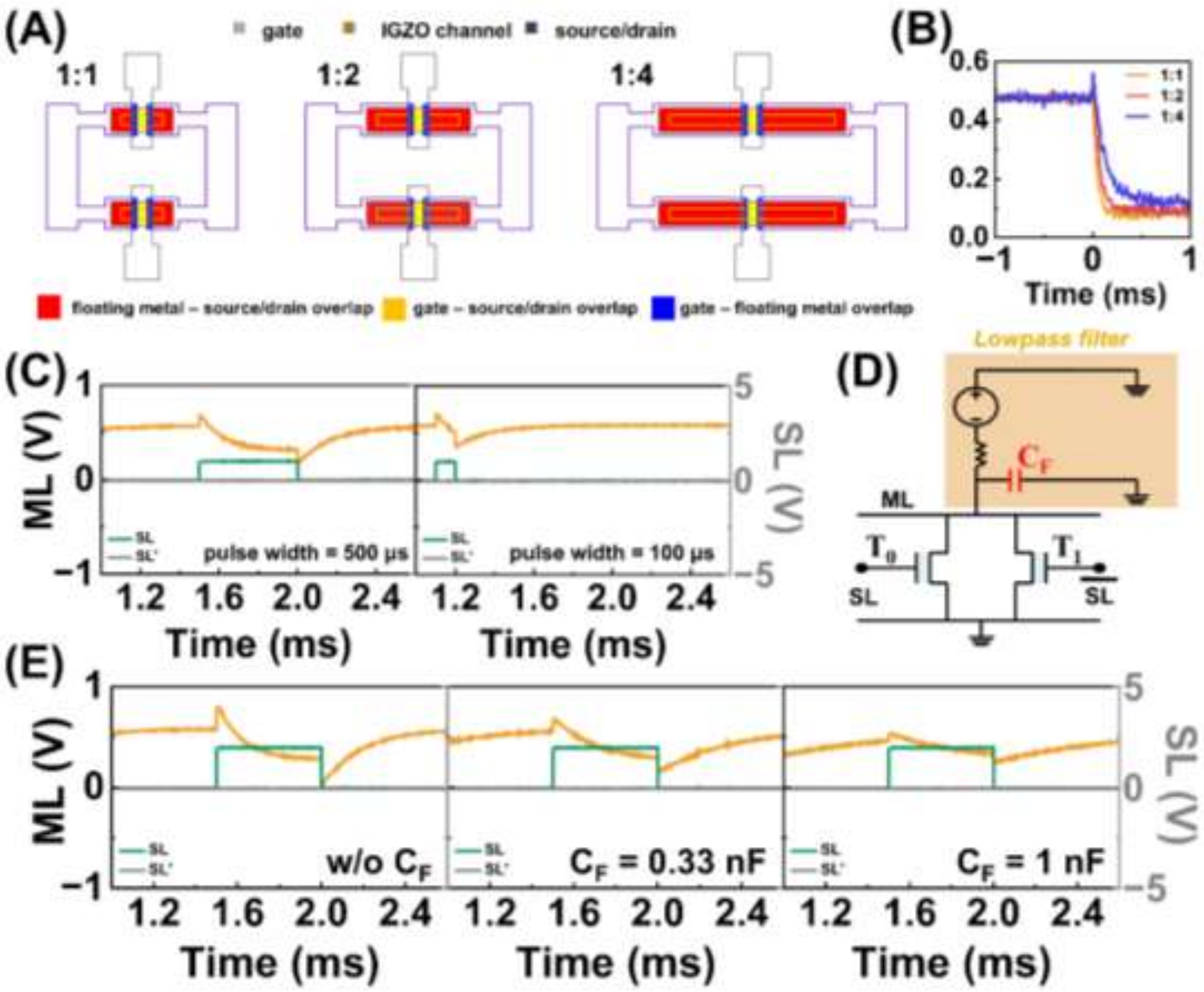

Figure S9. Analysis of the transient ML-voltage fluctuation during TCAM search operation. **(A)** Layouts of the 2FeTFT TCAM cells with MFM:MIS area ratios of 1:1, 1:2, and 1:4, highlighting the major capacitive-overlap regions, including the floating-metal–source/drain, gate–source/drain, and gate–floating-metal overlaps. The floating-metal–source/drain overlap area increases with increasing area ratio, resulting in increased capacitive loading on the ML. **(B)** Measured ML transient responses for the three area ratios. Increasing the area ratio results in a progressively slower ML charging/discharging response, consistent with an increased ML RC time constant, whereas the initial voltage-fluctuation amplitude remains comparatively insensitive to the area ratio. This indicates that the area-dependent device overlap capacitance contributes primarily to the ML settling dynamics but is not the sole origin of the transient fluctuation. **(C)** ML transient responses measured with search-pulse widths of 500 and 100 μs. At the shorter pulse width, the initial fluctuation and subsequent settling occupy a substantial

fraction of the search period, whereas a sufficiently settled ML level is obtained for the 500 μs pulse. **(D)** Circuit diagram illustrating the introduction of an additional filter capacitance, C_F, at the ML to suppress high-frequency transient components through low-pass filtering. **(E)** ML transient responses measured with = 0, 0.33, and 1 nF. Increasing C_F progressively suppresses the initial voltage fluctuation, while increasing the ML settling time, confirming the capacitive origin of the high-frequency transient component and the associated trade-off between fluctuation suppression and RC delay. Based on these results, a search-pulse width of 1 ms was employed in the TCAM measurements to provide sufficient settling time, and the sensing margin was evaluated after the initial transient had relaxed.

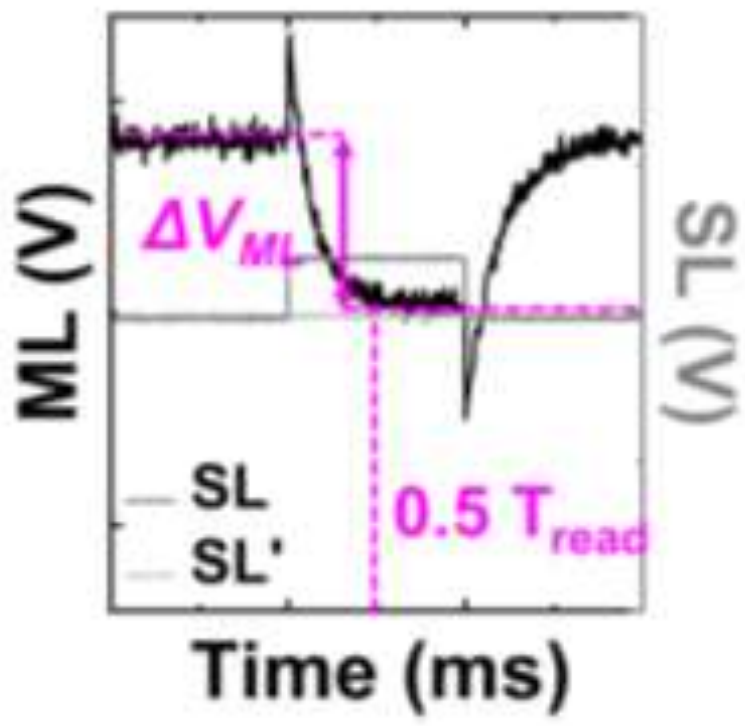

Figure S10. Definition of ΔV_{ML} during the TCAM search operation. The match line (ML) voltage transient is monitored under a read pulse, and ΔV_{ML} is defined as the voltage drop from the reference level at 50 % of the read-pulse duration (0.5 T_{read})

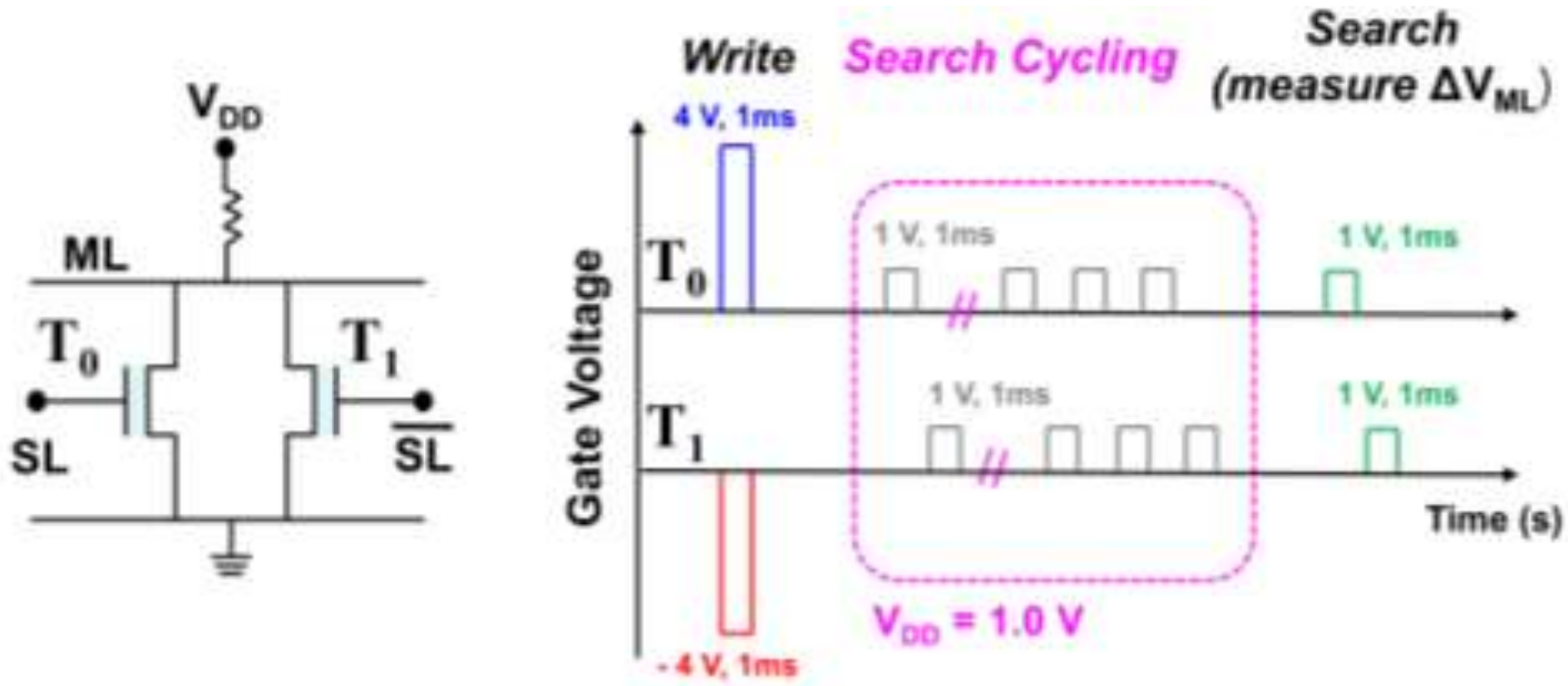

Figure S11. Schematic of the TCAM cell and pulse sequence used for the ΔV_{ML}-versus-search-cycle measurement. T0 and T1 were initially programmed using +4 V and −4 V, 1 ms write pulses, respectively. Search cycling was then performed using repeated 1 V, 1 ms gate pulses, while VDD = 1.0 V was continuously applied to the ML/drain side through the load resistor throughout the entire cycling period to emulate the actual TCAM search condition. At logarithmically spaced cycle numbers, ΔV_{ML} was evaluated using a 1 V, 1 ms search pulse.

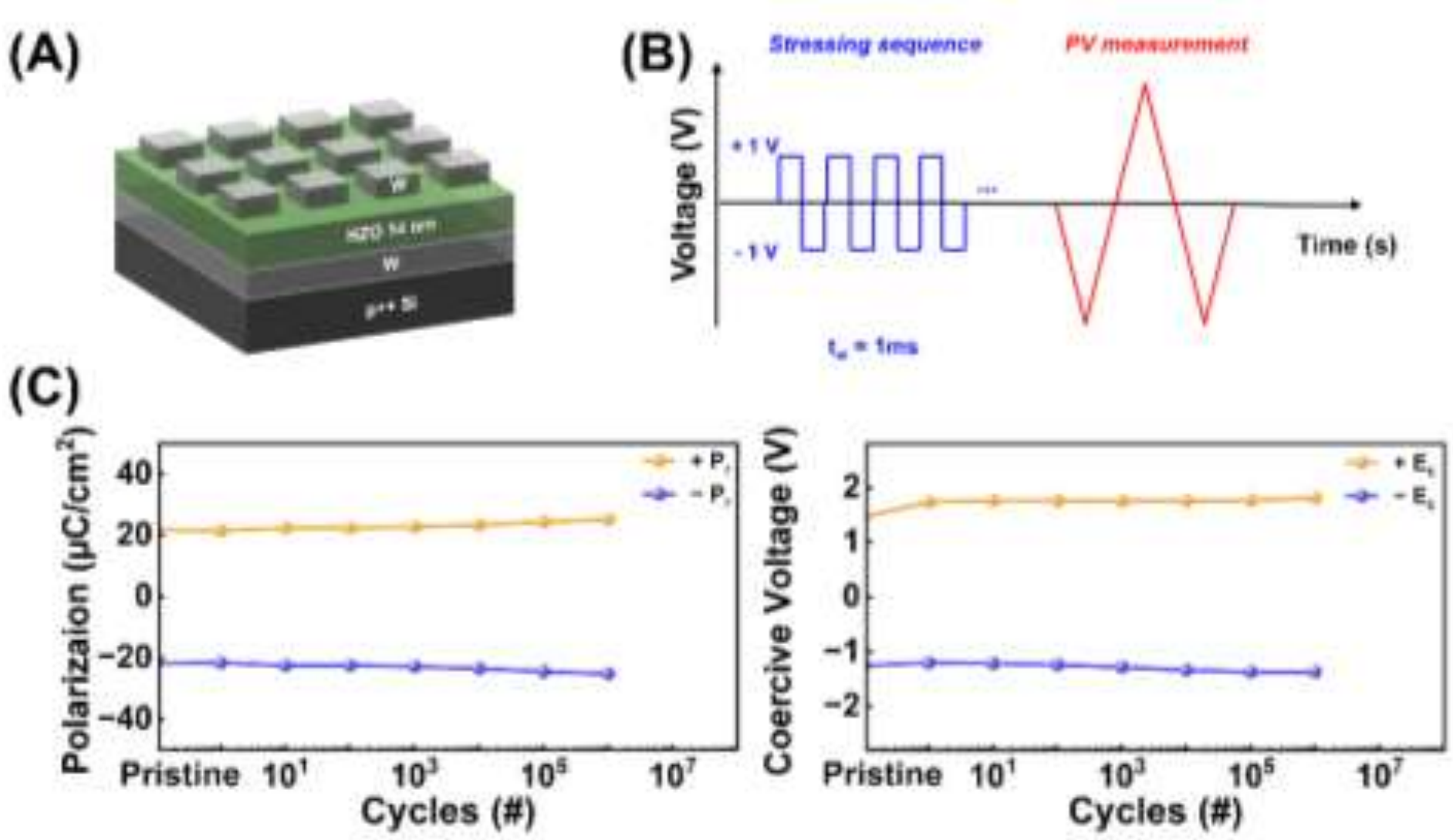

Figure S12. Search-equivalent cycling characteristics of the W/HZO (14 nm)/W MFM capacitor. (A) Schematic illustration of the MFM capacitor structure fabricated using the same HZO deposition process as that employed in the FeTFT. (B) Pulse sequence used for the cycling measurement. Bipolar ±1 V pulses with a pulse width of 1 ms were repeatedly applied, followed by P–V measurements at selected cycle numbers. Because the entire applied voltage is dropped across the ferroelectric stack in the standalone MFM capacitor, this condition represents a conservative upper-bound electrical stress relative to that experienced by the HZO layer during MFMIS FeTFT search operation. (C) Evolution of the remanent polarization + P_r and − P_r and coercive voltage + V_c and − V_c as a function of cycling up to 10^6 cycles, showing no pronounced wake-up or fatigue behavior.

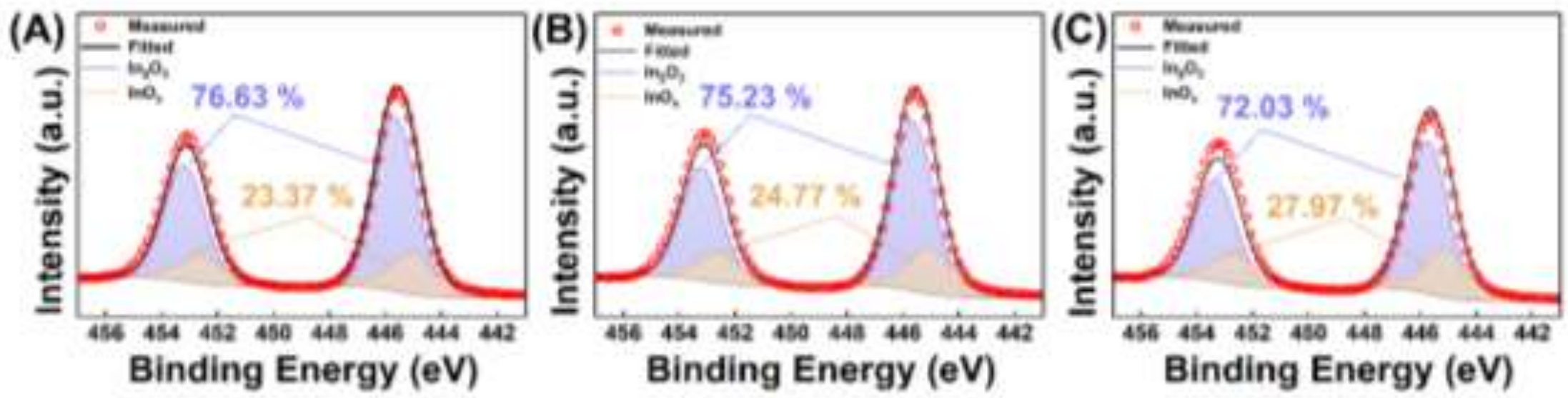

Figure S13. Deconvoluted In 3d XPS spectra of IGZO with (A) ZrO_2 11 nm, (B) ZrO_2 8 nm, and (C) HfO_2 8 nm gate dielectrics. The spectra were fitted with components corresponding to normal In–O bonding (In_2O_3) and oxygen-vacancy-associated In (InO_x). The extracted InO_x fractions were 23.37 %, 24.77 %, and 27.97 % for the ZrO_2 11 nm, ZrO_2 8 nm, and HfO_2 8 nm conditions, respectively.

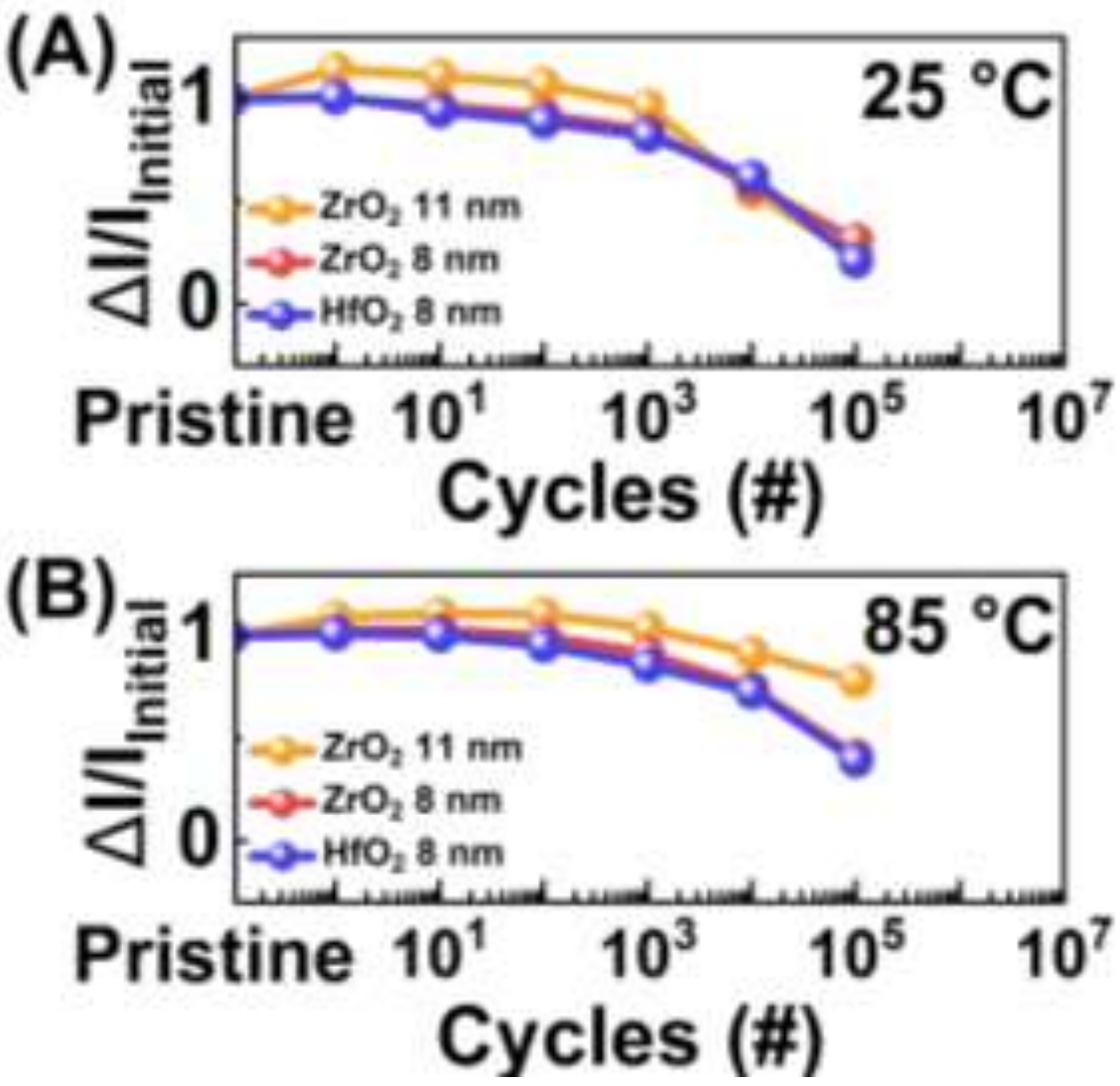

Figure S14. Temperature-dependent search-equivalent cycling characteristics of MFMIS FeTFTs with ZrO_2 11 nm, ZrO_2 8 nm, and HfO_2 8 nm gate dielectrics measured at (A) 25 °C and (B) 85 °C. Search-equivalent stressing was performed using repeated 1 V, 1 ms gate pulses under V_{DS} = 0.5V, reproducing the simultaneous vertical and lateral electric-field conditions relevant to TCAM search operation. The read current was extracted at V_{GS} = 0.5 V and V_{DS} = 0.5 Vand normalized to the corresponding pristine value. At 25 °C, an initial current increase is primarily observed for the ZrO_2 11 nm device, whereas at 85 °C, all three devices exhibit a slight initial current increase followed by progressive degradation with continued cycling.

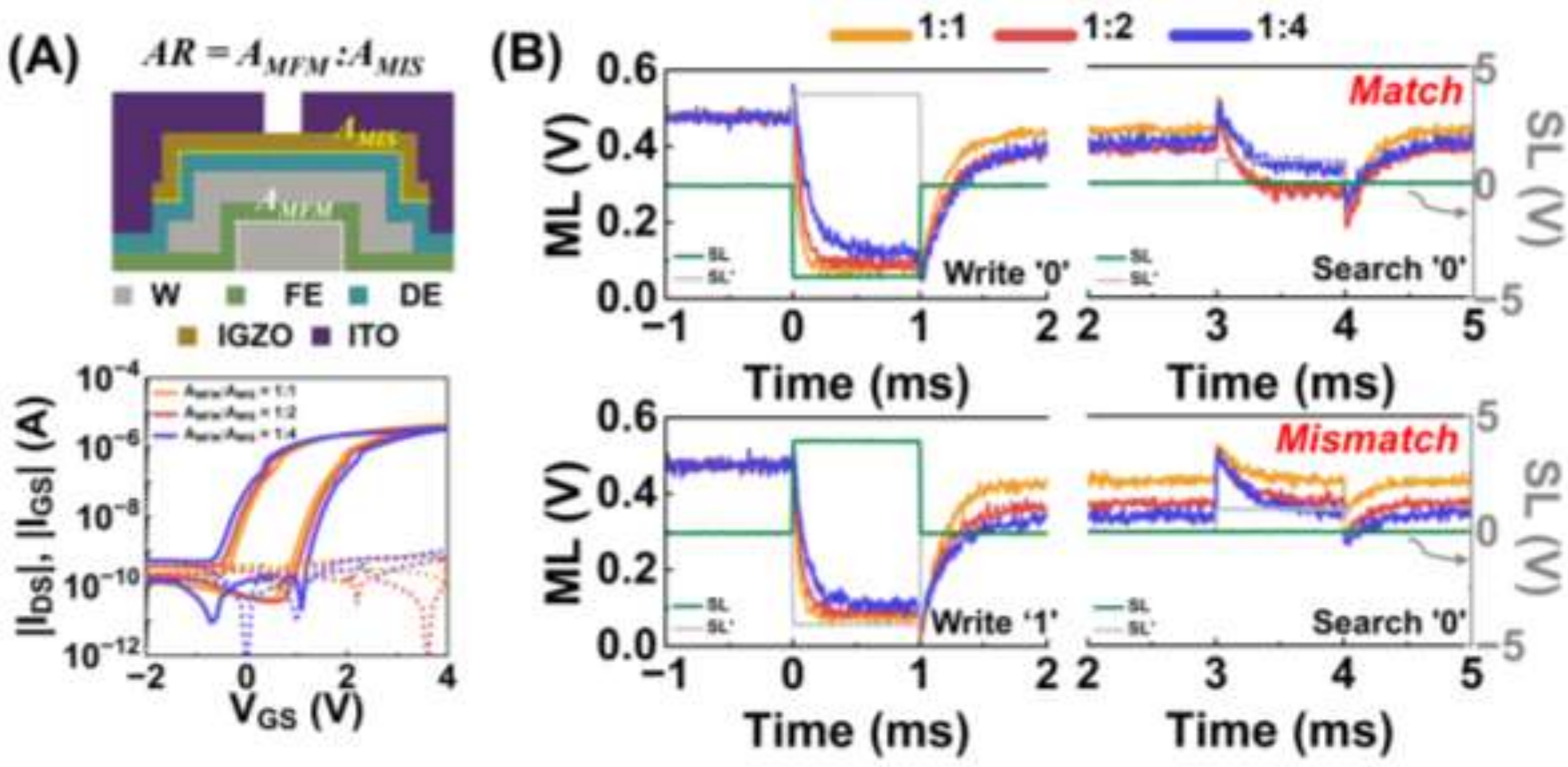

Figure S15. Effect of area ratio on memory window and TCAM sensing performance in MFMIS FeTFT-based 2T TCAM cells. (a) (Top) Schematic cross-section of the MFMIS FeTFT illustrating the area ratio (AR) definition AR = A_{MFM}:A_{MIS}, where A_{MFM} and A_{MIS} denote the areas of the MFM and MIS capacitors, respectively. (Bottom) Transfer characteristics for three area ratios (A_{MFM}:A_{MIS} = 1:1, 1:2, and 1:4), showing robust counterclockwise memory windows. (b) Time-domain matchline (ML) voltage waveforms for write and search operations across all three area ratio devices.

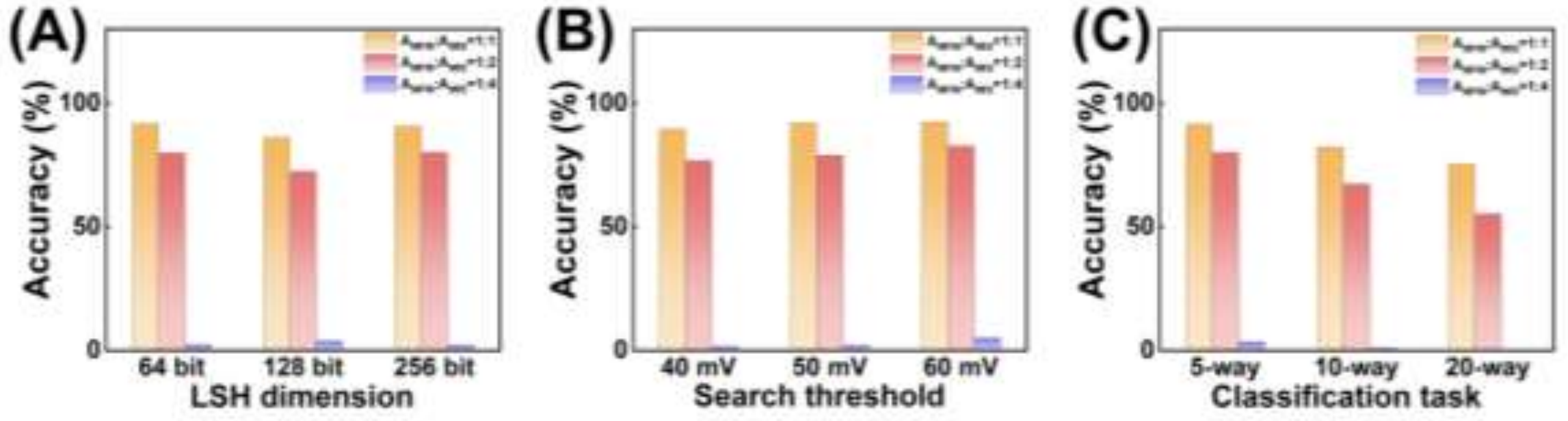

Figure S16. Simulation results of one-shot learning classification accuracy for the three area ratio devices under varying hardware parameters. (a) Accuracy as a function of LSH dimension (64, 128, 256 bit), (b) accuracy versus search threshold voltage (40, 50, 60 mV), and (c) accuracy across classification task complexity (5-way, 10-way, 20-way). The 1:1 area ratio consistently achieves the highest accuracy, while the 1:4 ratio shows degraded performance attributed to the reduced sensing margin arising from asymmetric polarization distribution. Although increasing the area ratio enlarges the memory window by providing more favorable voltage distribution for ferroelectric switching, it simultaneously increases parasitic capacitance and RC delay of the match line, resulting in progressively slower ML transient response and reduced ΔV_{ML} at a given sensing time. These results highlight a fundamental trade-off between ferroelectric switching characteristics and circuit-level sensing performance, demonstrating that area ratio optimization must account for both storage and dynamic response requirements in TCAM operation.

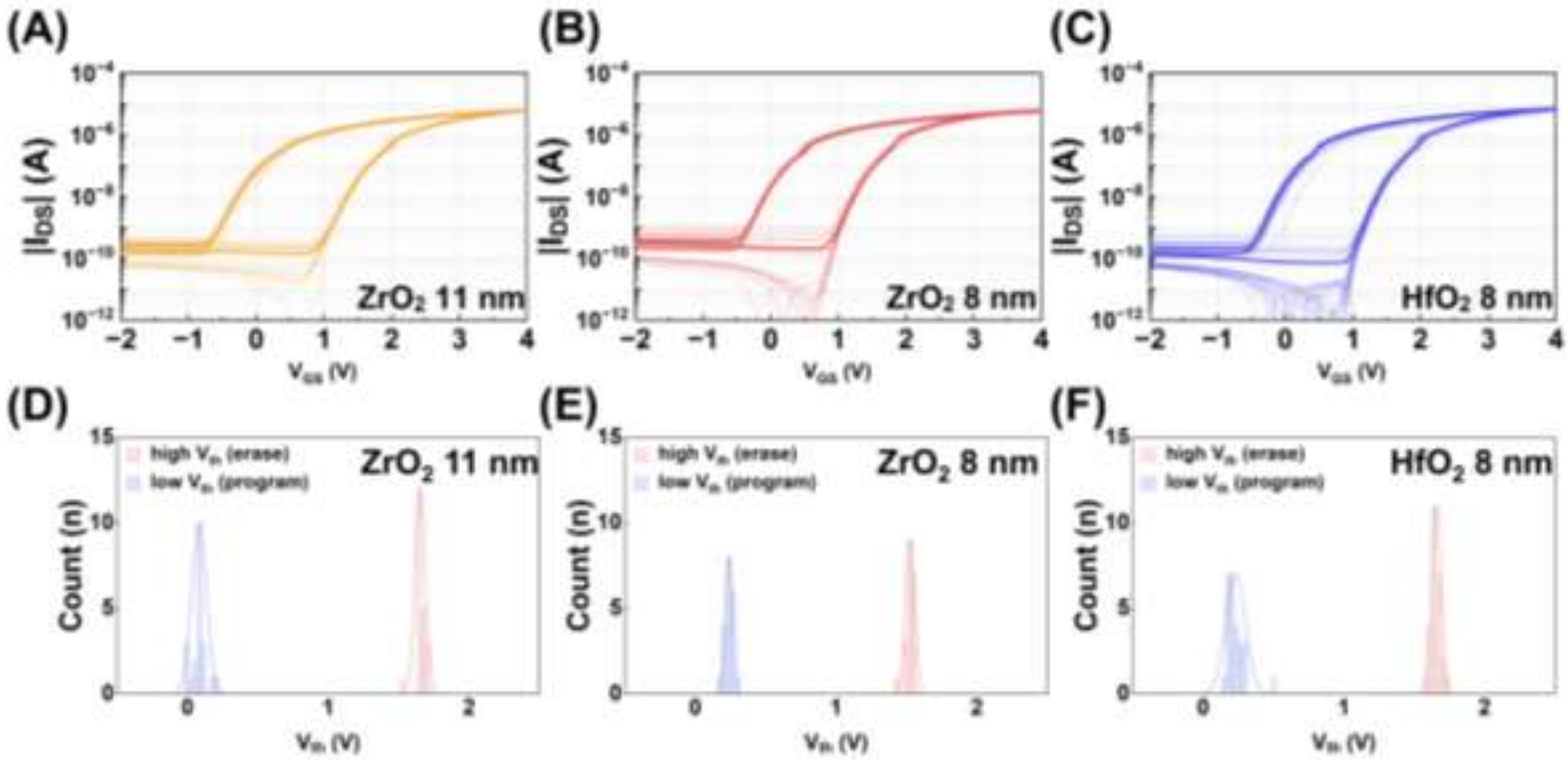

Figure S17. (A-C) Transfer characteristics of the ZrO_2 11 nm, ZrO_2 8 nm, and HfO_2 8 nm devices, respectively. (D-F) Statistical distributions of the extracted high-V_{th} (erase) and low-V_{th} (program) states for the corresponding devices. The results confirm stable separation between the programmed and erased states for all three dielectric conditions. The threshold-voltage distributions were obtained from 30 devices and fitted with Gaussian functions, and the extracted standard deviations were subsequently used as the sigma values in the simulation.

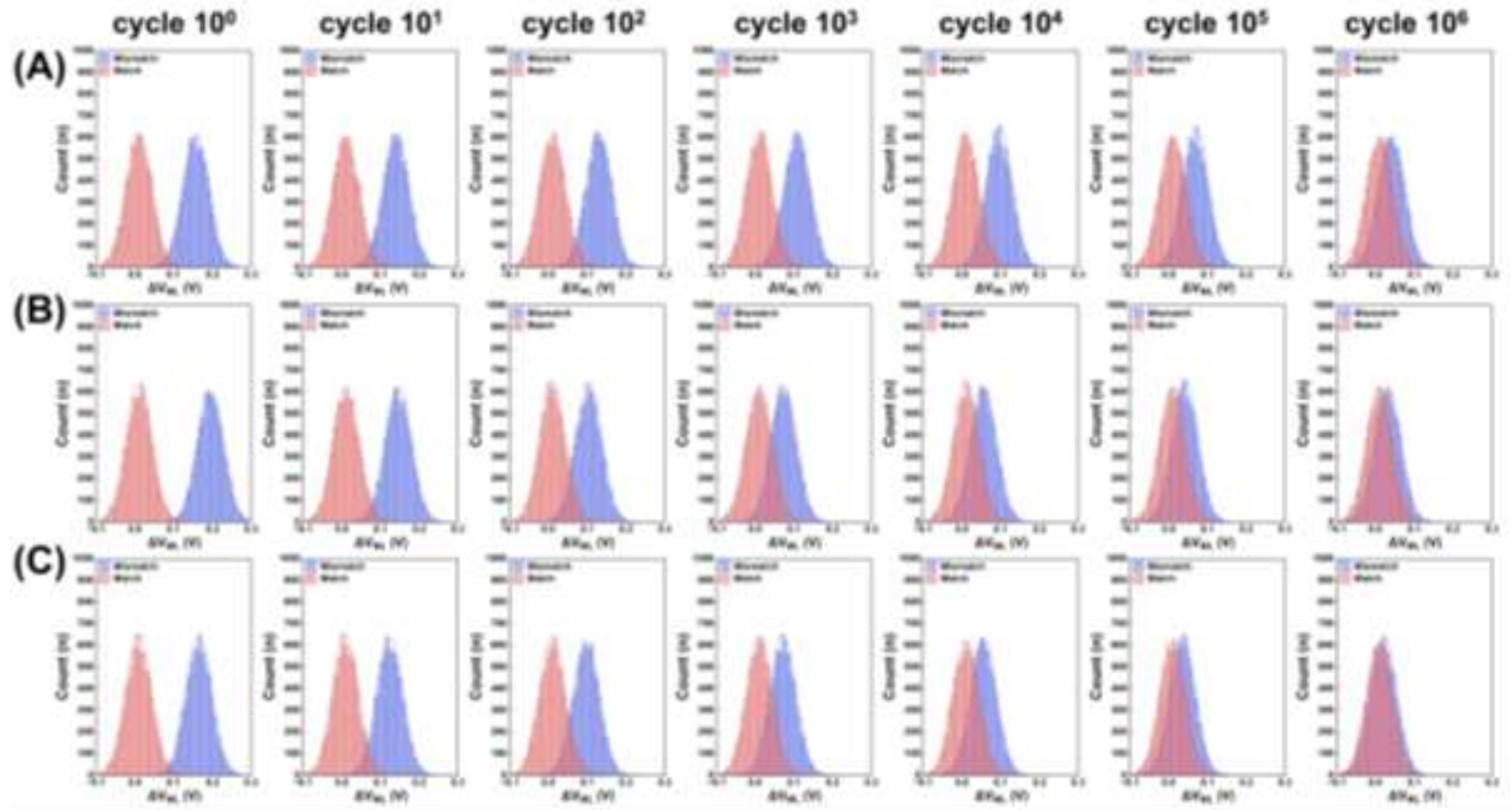

Figure S18. Monte Carlo-generated distributions of ΔV_{ML} for the match and mismatch states as a function of search cycle number, used in the TCAM-based one-shot classification simulation. Results for the (a) ZrO_2 11 nm, (b) ZrO_2 8 nm, and (c) HfO_2 8 nm devices, respectively. For each device and cycle condition, the experimentally measured mean ΔV_{ML} values of the match and mismatch states were used as the centers of the corresponding Gaussian distributions, while the standard deviation extracted from the single-device statistical analysis was used as the effective σ in the simulation. Based on these parameters, 10,000 ΔV_{ML} samples were generated for each state by Monte Carlo sampling to emulate the statistical variation of the TCAM-cell sensing signal. The resulting sampled distributions were then used to estimate sensing error probability, from which the bit-error rate and classification accuracy were calculated in the subsequent one-shot learning simulation.
